# Apical Transport of Influenza A Virus Ribonucleoprotein Requires Rab11-positive Recycling Endosome

**DOI:** 10.1371/journal.pone.0021123

**Published:** 2011-06-22

**Authors:** Fumitaka Momose, Tetsuya Sekimoto, Takashi Ohkura, Shuichi Jo, Atsushi Kawaguchi, Kyosuke Nagata, Yuko Morikawa

**Affiliations:** 1 Kitasato Institute for Life Sciences, Kitasato University, Tokyo, Japan; 2 Graduate School of Comprehensive Human Sciences, University of Tsukuba, Tsukuba, Ibaraki, Japan; Institut National de la Santé et de la Recherche Médicale, France

## Abstract

Influenza A virus RNA genome exists as eight-segmented ribonucleoprotein complexes containing viral RNA polymerase and nucleoprotein (vRNPs). Packaging of vRNPs and virus budding take place at the apical plasma membrane (APM). However, little is known about the molecular mechanisms of apical transport of newly synthesized vRNP. Transfection of fluorescent-labeled antibody and subsequent live cell imaging revealed that punctate vRNP signals moved along microtubules rapidly but intermittently in both directions, suggestive of vesicle trafficking. Using a series of Rab family protein, we demonstrated that progeny vRNP localized to recycling endosome (RE) in an active/GTP-bound Rab11-dependent manner. The vRNP interacted with Rab11 through viral RNA polymerase. The localization of vRNP to RE and subsequent accumulation to the APM were impaired by overexpression of Rab binding domains (RBD) of Rab11 family interacting proteins (Rab11-FIPs). Similarly, no APM accumulation was observed by overexpression of class II Rab11-FIP mutants lacking RBD. These results suggest that the progeny vRNP makes use of Rab11-dependent RE machinery for APM trafficking.

## Introduction

The viral genomes do not exist alone but form nucleoprotein complexes in which DNA/RNA genome is complexed with viral basic proteins, e.g., nucleocapsid protein for retrovirus [1] and core protein VII for adenovirus [2], [3]. In the case of influenza A virus, a member of *Orthomyxoviridae*, a virion contains eight distinct segments of viral/virion ribonucleoprotein complexes (vRNPs) and each vRNP segment consists of a single-stranded negative-sense virion RNA (vRNA), viral RNA-dependent RNA polymerase (heterotrimer of PB2, PB1, and PA subunits), and nucleoprotein (NP) [4]. Both 5′ and 3′ termini of vRNA segment form a partially complementary double-stranded structure called “panhandle” [5], [6] and function as promoter and replication origin for viral RNA synthesis. The viral RNA polymerase primarily binds to the panhandle region, whereas NP binds to the single-stranded region [7], [8], [9], [10]. During viral genome replication, complementary RNA (cRNA) segments are synthesized from vRNA segments and progeny vRNAs are further amplified from the cRNA segments. Although both cRNA and progeny vRNA form viral RNP complexes, it has been shown that cRNP only localizes in the nucleus [11], [12].

Trafficking of viral genome-nucleoprotein complex from the cell surface to sites of viral genome replication involves cellular trafficking machineries [13], [14]. Some viruses, e.g., HIV-1 and herpes simplex virus (HSV), fuse with the plasma membrane and their nucleoprotein complexes ride on “tracks” such as actin filaments and microtubules [15], [16]. Other viruses, e.g., Semliki Forest virus, adenovirus, and influenza virus, taken up by endocytosis [17], [18], [19], [20], might be transported on the cytoskeletal tracks in the cytoplasm. In the case of influenza virus, trafficking of endocytosed virions to the perinuclear region has been visualized by live cell imaging [21]. It is well known that endocytosed influenza virus is uncoated at low pH endosomes and vRNP segments are relocated into the nucleus where replication of influenza virus genome occurs [22].

Newly synthesized nucleoprotein forms a complexes with viral genome and the complex is transported to sites of genome packaging/virion budding: the apical plasma membrane (APM) of polarized epithelial cells for influenza virus and respiratory syncytial virus (RSV) [23], [24]; the basolateral plasma membrane for vesicular stomatitis virus [23], [25]; intracellular membranes for herpes viruses [26]. Like viral entry, these viral egress pathways depend on cytoskeletons, transport vesicles, and/or motor proteins [27]. To identify such transport pathways utilized for incoming and outgoing viruses, a number of organelle marker proteins, e.g., EEA1, mannose 6-phosphate receptors, LAMP1, and small GTPase Rab family proteins are used [28], [29]. Progeny viruses were finally released from cells by cell lysis or membrane budding followed by pinching-off. The endosomal sorting complex required for transport (ESCRT) machinery, especially ESCRT-III and VPS4 were often used for release of some viruses such as retroviruses [30], [31]. However, other viruses require additional release machinery (e.g., prototype foamy virus and parainfluenza virus 5) [32], [33], [34] or do not require the ESCRT machinery (e.g., RSV and influenza virus) [35], [36], [37]. It has been reported that release of RSV is independent of ESCRT machinery but controlled by Rab11 family interacting protein 2 (Rab11-FIP2), an effector protein of Rab11 [35]. Similarly, influenza virus particle budding and filamentous viral formation are controlled by the Rab11 system including a related factor(s) such as Rab11-FIP3 [38].

To elucidate these trafficking pathways for outgoing viruses, live cell imaging has been employed and revealed that microtubules are tracks for egress of vaccinia virus [39], [40], [41]. Single-stranded RNA virus genomes (e.g., poliovirus and RSV) have also been visualized with fluorescent antisense nucleotide probes called molecular beacon in living cells [42], [43]. However, such viral genomes are complexed with viral nucleoproteins, which are the essence of viral infectivity, but nevertheless is poorly delineated because of a lack of specific detection system. We obtained anti-NP monoclonal antibody (mAb61A5) that preferentially bound to influenza viral RNP complexes rather than free NP and found that progeny viral RNP complexes distributed as punctate signals and concentrated at the microtubule organizing center (MTOC) in fixed cells [44]. Fluorescence *in situ* hybridization (FISH) assays confirmed that the punctate RNP signals contained negative-sense viral RNA [45]. Here, we report that progeny vRNPs of influenza virus primarily target to the small GTPase Rab11-positive recycling endosome (RE), also known as endocytic recycling compartment (ERC), through interaction between an active/GTP-bound Rab11 molecule(s) and a heterotrimeric viral RNA-dependent RNA polymerase of vRNP. Our data also indicate that the targeting to RE is required for the cytoplasmic trafficking of vRNP to the APM along microtubules and subsequent virion production. Based on our data and others, we propose a model for a higher-order assembly of vRNP segments toward virion packaging.

## Results

### Live cell imaging of progeny vRNP in the cytoplasm

Our previous studies with paraformaldehyde-fixed cells found the potential of anti-NP mAb61A5 for detection of the vRNPs in the cytoplasm of influenza virus infected cells [44], [45]. Anti-NP mAb61A5 preferentially bound to influenza viral RNP complexes and immunostaining using this antibody showed punctate NP antigens in the cytoplasm after 4 hours postinfection (hpi). Further FISH analysis revealed that the punctate NP antigen contains viral genome RNAs. These punctate signals of vRNPs were localized along the microtubules and later accumulated at the APM. Depolymerization of microtubules by nocodazole dispersed the punctate vRNP signals in the cytoplasm, suggesting microtubule-dependent transport of progeny vRNPs.

**Figure 1:**
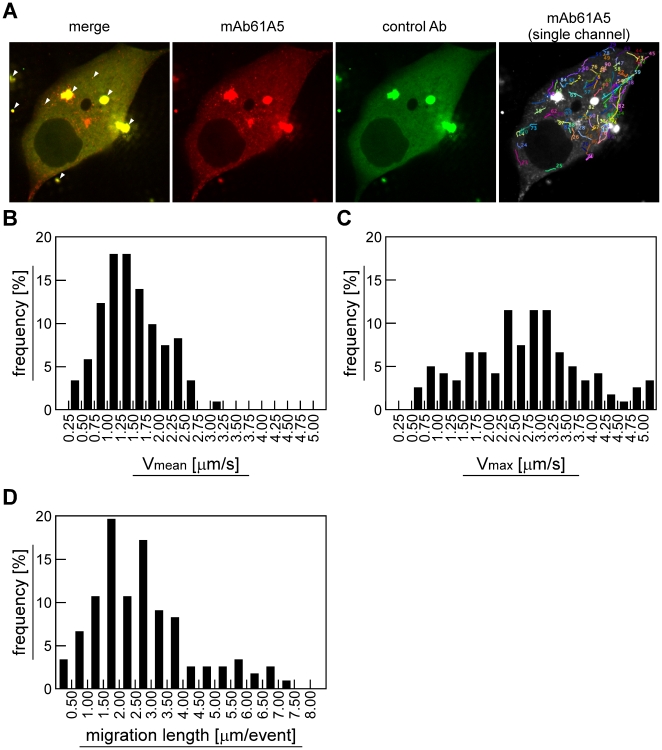
Live cell imaging of cytoplasmic vRNPs in infected MDCK cells. (**A**) For live cell imaging, AF568-conjugated anti-NP mAb61A5 (red, mAb61A5) and AF488-conjugated non-specific mouse immunoglobulin (green, control Ab) were cotransfected to infected MDCK cells. Sequential images were acquired by the dual-color protocol and subsequently by the single-color protocol for kinetic analysis. Images were processed and analyzed by using ImageJ software and MTrackJ plugin (Video S1). A representative frame of the movie was shown (left 3 images). Pseudo-positive signals appeared in yellow in merged image (most left image, arrowheads). An example of signal tracking was shown as trajectories (most right image, mAb61A5 single channel). (**B** and **C**) Velocity distribution of vRNP signals. Mean and maximum velocities (V_mean_ and V_max_, respectively) of individual motile events were calculated and shown as histograms (Table S7, total 123 motile events derived from 75 trajectories). (**D**) Distribution of migration lengths. The migration lengths of individual motile events were shown as a histogram.

To understand dynamic events of progeny vRNP, here we carried out live cell imaging of vRNP signals (Figure 1A). To this end, fluorescent-labeled mAb61A5 was introduced into infected cells with protein transfection reagents. Dual-color imaging of mAb61A5 (Figure 1A, red) and non-specific control antibody (Figure 1A, green) eliminated pseudo-positive signals, likely corresponding to aggregates of antibodies and non-specifically endocytosed antibodies upon liposome-mediated transfection (Figure 1A, arrowheads, yellow in merged image) and allowed us to detect true outgoing vRNP signals (red alone in merged image). Live cell imaging revealed that the vRNP signals moved rapidly but intermittently in both forward and backward directions (Figure 1A and Video S1). We defined one motile event as a single unidirectional movement (see Materials and Methods). Tracking of vRNP signals showed that 72% of mean velocities (V_mean_) of individual motile events were ranged from 0.75 to 2.00 µm/s and the mean overall V_mean_ was 1.45 µm/s (Figure 1B and Table S7). This mean velocity is likely to correspond to a microtubule- and motor protein-dependent vesicular transport, since it has been reported that KIF1A particles moved in axons anterogradely at 1.00±0.61 µm/s and sometimes retrogradely at 0.72±0.27 µm/s [46], (see the discussion). Some of the maximum velocities (V_max_) observed in individual events reached over 5.00 µm/s (Figure 1C). Mean of migration lengths of individual events was 2.68 µm and the maximum length reached 7.48 µm (Video S1 and Table S7, trajectory No. 5, during 14.00 to 18.25 s). Mock-infected MDCK cells with heat-inactivated virus did not show any vRNP-specific signals but only pseudo-positive signals (Video S2, left half).

**Figure 2:**
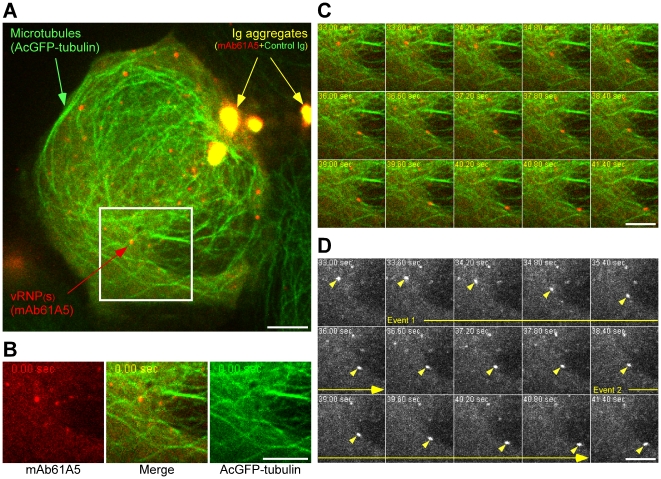
Live cell imaging of cytoplasmic vRNPs along microtubules. (**A**) Live cell imaging was carried out using MDCK cells expressing AcGFP-α-tubulin. Pseudo-positive signals (yellow), the microtubule networks (green), and vRNPs (red) were indicated as arrows. (**B**) Cropped and each color-split image of the indicated area (white box in panel A). Sequential images were shown in Video S3. (**C** and **D**) Time-split images of the merged images and the mAb61A5 channel images in the cropped area, respectively. Elapsed time from the first acquisition was indicated on each image. A vRNP signal (arrowheads in D) moved (event 1, 33.6 to 36.0 s), paused (36.0 to 38.4 s), and moved again (event 2, 38.4 to 40.8 s). Scale bars  = 5 µm.

To analyze whether vRNP signals move along microtubules, we established an AcGFP-α-tubulin expressing MDCK cell line (MDCK-Tub) and carried out dual-color imaging (Figure 2). Progeny vRNP signals localized to (Figure 2, panels A and B) and moved along microtubules (Figure 2C and Video S3). A vRNP signal (Figure 2D, arrowheads) often moved intermittently: (i) pausing (0.0 to 33.6 s), (ii) moving (event 1, 33.6 to 36.6 s, duration of 3.0 s), (iii) pausing again (36.6 to 38.4 s), and (iv) moving again (event 2, 38.4 to 41.4 s, duration of 3.0 s). These observations indicated that progeny vRNPs are transported through the microtubule-dependent trafficking machinery.

### Progeny vRNPs are colocalized with Rab11-positive compartments in the cytoplasm

**Figure 3:**
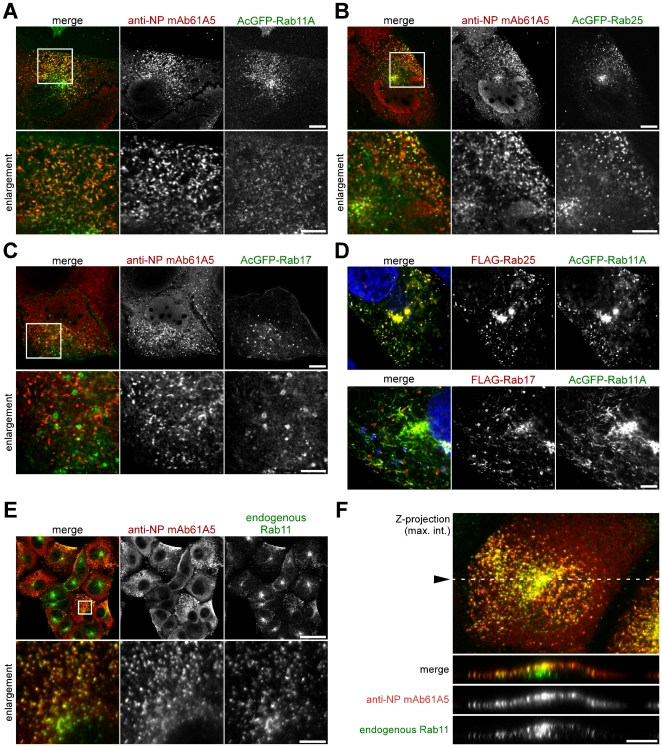
Colocalization of punctate vRNP signals with Rab11. (**A–C**) Localizations of cytoplasmic vRNPs and transiently expressed human Rab proteins. Influenza A virus was infected to MDCK cells transiently expressing AcGFP-tagged human Rab11A, Rab25, and Rab17 (panels A–C, respectively). At 7 hpi, vRNPs were immunostained with mAb61A5 (center image in each set) and visualized by confocal microscopy with AcGFP-Rab proteins (right images). Enlarged images of indicated areas (white boxed) were also shown (lower images). Scale bars are 10 and 5 µm (upper and lower images, respectively). (**D**) Localizations of transiently expressed human Rab11A, Rab25, and Rab17. FLAG-Rab25 (upper) and FLAG-Rab17 (lower) (center images) were coexpressed with AcGFP-Rab11A (right images) in MDCK cells. Nuclei were stained with DAPI (blue, left images). Scale bar is 5 µm. (**E** and **F**) Colocalization of vRNP with endogenous Rab11. Progeny vRNPs were similarly stained with mAb61A5. Endogenous canine Rab11 (right images) was visualized with rabbit anti-Rab11 polyclonal antibody. (E) XY presentation. Scale bars are 40 and 5 µm (upper and lower images, respectively). (F) XZ presentation. Z-stacks of confocal images were acquired at 0.5 µm z-axis interval. Z-projection of maximum intensities (top image) and reconstitution of a xz plane (lower 3 images) were processed by using ImageJ software. Dotted line indicates the position of the reconstituted xz plane. Scale bar is 10 µm.

We have previously reported that the vRNP signals were colocalized with microtubules and concentrated at the MTOC [44]. Given the fact that cytoplasmic vesicles are often accumulated at the MTOC and are transported on microtubules [14], our data suggest that the vRNPs were able to be transported on vesicles. Indeed, the behavior of vRNP signals we observed by live cell imaging (Figures 1 and 2, Videos S1 and S3) resembled that of Rab10, small GTPase protein involved in vesicle trafficking [47]. Based on these hypotheses, we carried out identification of cytoplasmic compartments involved in vRNP trafficking by immunofluorescence microscopy. We constructed 20 distinct classes of Rab proteins as markers for transport vesicles, all of which were tagged with AcGFP (Table S1, except for Rab11B). Each of Rab family proteins is implicated in distinct vesicle trafficking [28], [29]. We assessed the colocalization with vRNPs by confocal microscopy: AcGFP-Rab11A was almost completely, and AcGFP-Rab25 and -Rab17 were partially colocalized with vRNP signals (Figure 3, panels A, B, and C, respectively). The others we tested did not show significant colocalizations with vRNP signals (data not shown). Since Rab11A [48], [49], [50], Rab25 [51], [52], and Rab17 [53], [54] are known as marker proteins of RE, our results suggested that the progeny vRNP segments were transported via RE.

Although these three Rab proteins participate in RE trafficking [28],[29], their precise distributions may differ from each other. We coexpressed either FLAG-Rab25 or FLAG-Rab17 with AcGFP-Rab11A in MDCK cells and observed their localizations (Figure 3D). The majority of FLAG-Rab25 was colocalized with AcGFP-Rab11A (Figure 3D, upper images), whereas FLAG-Rab17 was rarely colocalized with AcGFP-Rab11A except for the perinuclear region, which may correspond to the pericentriolar ERC/RE (Figure 3D, lower images). From these results, we reasoned that progeny vRNPs might target and accumulate to the Rab11-positive RE in the cytoplasm.

We verified the colocalization of cytoplasmic vRNP with endogenous Rab11. Confocal imaging revealed that vRNPs colocalized with endogenous Rab11 at the cell periphery of MDCK cells at 7 h postinfection (hpi) (Figure 3E). The fluorescent image of xz plane reconstituted from the image stack showed that, at this time point, the majority of progeny vRNPs were colocalized with Rab11 and both were accumulated at the upper cell surface (Figure 3F), although a fraction of endogenous Rab11 remained at the perinuclear region.

### Active/GTP-bound Rab11 is required for localization of progeny vRNP to RE

**Figure 4:**
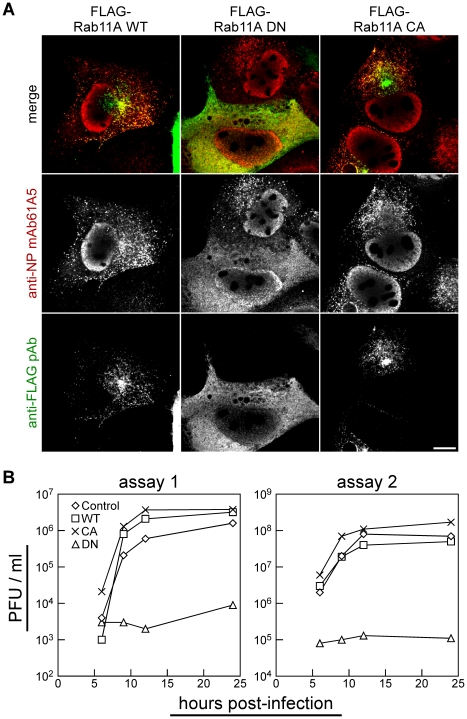
Localization of progeny vRNPs to RE in active/GTP-bound Rab11 dependent manner. (**A**) Alteration of vRNP localization by transient expression of dominant negative Rab11 mutant. Influenza A virus was infected to MDCK cells transiently expressing the wild type (WT, left images), dominant negative (DN, center images), and constitutively active (CA, right images) forms of FLAG-tagged human Rab11A. At 7 hpi, vRNPs (middle images) and FLAG-Rab proteins (bottom images) were immunostained using mAb61A5 and rabbit anti-FLAG polyclonal antibody (pAb) and observed by confocal microscopy. Scale bar is 10 µm. (**B**) Production of infectious progeny viruses from infected MDCK cells constitutively expressing human Rab11A and its mutants. Culture supernatants of MDCK cells infected with PR8 strain at moi = 1 to 3 were temporally harvested and titers of infectious viruses were measured and indicated as plaque forming unit (pfu)/ml. Single-round infection experiments were carried out using different lots of viral inoculum in independent experiments.

The small GTPase Rab family protein is activated upon GTP binding and is inactivated by GTP hydrolysis [55], [56]. The single-point mutations around the GTPase active site, i.e., substitutions of the serine residue at amino acid position 25 to an asparagine residue (S25N) or the glutamine residue at amino acid position 70 to a leucine residue (Q70L), have been shown to stabilize the Rab11A protein in GDP- or GTP-bound states [49]. To test whether expression of GDP/GTP-locked Rab11 affects the localization of vRNP to RE, we constructed dominant negative (designated DN, S25N substitution) and constitutively active (designated CA, Q70L substitution) mutants of FLAG-Rab11A and expressed in MDCK cells. Transient expression of CA Rab11A did not alter the localization of progeny vRNP signals to RE (Figure 4A, right images) but, in contrast, expression of the DN Rab11A markedly impaired the localization to RE, showing that vRNP was diffusely distributed throughout the cytoplasm (Figure 4A, center images). Essentially similar results were observed when Rab11B and its mutants were used (data not shown). These results indicate that progeny vRNP targeting and/or localization to RE require active/GTP-bound Rab11.

Next, we examined their impacts on viral replication (Figure 4B). Influenza virus was infected to MDCK cell lines in which wild type (WT), DN mutant, and CA mutant of FLAG-Rab11A were constitutively expressed (MDCK-F11A-WT, -DN, and -CA, respectively), and infectious progeny viruses were titrated by plaque assays. No significant differences in the kinetics of infectious virus production were observed between MDCK-F11A-CA and -WT cells and even with MDCK cells containing the empty vector (MDCK-Neo). However, viral production in MDCK-F11A-DN cell line was severely impaired with a 99.0–99.9% reduction at 24 hpi. Together, these results indicate that the targeting of progeny vRNP to RE is necessary for trafficking of vRNP segments and subsequent efficient infectious virus production.

### vRNPs are coimmunoprecipitated with active/GTP-bound Rab11

**Figure 5:**
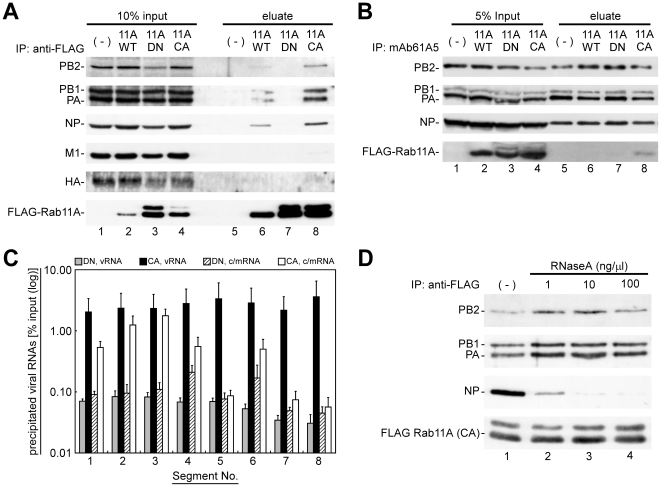
Coimmunoprecipitation of progeny vRNP segments with active/GTP-bound Rab11A. (**A**) Coimmunoprecipitation of viral proteins with FLAG-Rab11A and its mutants. MDCK-Neo (lanes 1 and 5), MDCK-F11A-WT (lanes 2 and 6), -DN (lanes 3 and 7), and -CA (lanes 4 and 8) cells were infected with PR8 strain and harvested at 7 hpi. PNS were subjected to immunoprecipitation assays using anti-FLAG mAb, and 10% input (lanes 1–4) and precipitates (lanes 5–6) were analyzed by Western blotting with mouse anti-HA antiserum and anti-FLAG mAb, rabbit anti-PB2, PB1, PA, NP, and M1 antisera. (**B**) Coimmunoprecipitation of FLAG-Rab11 CA mutant with viral RNP complexes. Immunoprecipitation assay was carried out using anti-NP mAb61A5. Precipitates were treated with RNase A and eluates were subjected to Western blotting analysis. (**C**) Coimmunoprecipitation efficiencies of viral RNAs. The amounts of viral RNAs in the immunoprecipitates with anti-FLAG mAb were quantified by polarity-specific reverse transcription followed by segment-specific semiquantitative real-time PCR. Coimmunoprecipitation efficiencies were calculated as percentage of RNA amounts in precipitates relative to those in the input (Figure S3). Segment numbers were indicated at the bottom. Columns indicated the coimmunoprecipitation efficiencies of vRNAs (gray and black columns) and c/mRNAs (hatched and white columns) from MDCK-F11A-DN and -CA. (**D**) Coimmunoprecipitation of vRNP components in the presence of RNase A. Immunoprecipitation assays using infected MDCK-F11A-CA cells were carried out in the absence (lane 1) or the presence of 1, 10, and 100 ng/µl RNase A (lanes 2–4, respectively). Coprecipitated vRNP components (PB2, PB1, PA, and NP) and direct precipitates (FLAG-Rab11A CA) were detected by Western blotting.

The interaction of vRNP with active/GTP-bound Rab11 was examined by immunoprecipitation (Figure 5A). MDCK-Neo, MDCK-F11A-WT, -DN, and -CA cells were infected with influenza virus and post-nuclear supernatants (PNS) were incubated with anti-FLAG mAb, and immunoprecipitated (Figure 5A, lanes 5–8). Western blotting analyses revealed that all protein components of vRNP (PB2, PB1, PA, and NP) were coimmunoprecipitated with the WT and CA mutant FLAG-Rab11A proteins (Figure 5A, lanes 6 and 8, respectively) but not with the DN mutant (Figure 5A, lane 7). Reversely, FLAG-Rab11 CA mutant was coprecipitated, when viral RNP complexes were immunoprecipitated by mAb61A5 (Figure 5B, lane 8). Other viral proteins, such as HA and M1, were not coimmunoprecipitated with FLAG-Rab11A proteins (Figure 5A). These results were in good agreement with our immunofluorescence observations that cytoplasmic HA signals did not colocalize with progeny vRNP signals, when detected by *in situ* hybridization [45] or by mAb61A5 (Figure S2). These results indicate that the transport vesicles for progeny vRNP segments are distinct from those for viral membrane/matrix proteins.

Next, we focused on classes of viral RNAs in the immunoprecipitate, namely vRNA segments with negative polarity and cRNA segments or mRNA with positive polarity (c/mRNA), and classes of RNA segments. We carried out polarity-specific reverse transcription followed by segment-specific semiquantitative real-time PCR (Figure 5C). All vRNA segments were coimmunoprecipitated with Rab11A CA mutant at relatively equal efficiency (2.0–3.6% of input vRNA, Figure S3). These precipitates were not observed with the DN mutant (less than 0.1% of input). The data suggest, although do not prove, that vRNA was coimmunoprecipitated as a component of vRNP and that the coimmunoprecipitation depended on a common characteristic of all vRNA segments, such as terminal panhandle structures rather than segment-specific base sequences or segment lengths. Some of c/mRNAs were also coimmunoprecipitated in an active/GTP-bound Rab11-dependent manner. The coimmunoprecipitation efficiencies of c/mRNAs likely depended on their base lengths to some extent.

### Viral heterotrimeric RNA polymerase is the primary component required for Rab11-vRNP interaction

To ascertain the primary component of vRNP required for the interaction with active/GTP-bound Rab11, we carried out immunoprecipitation assays in the absence or the presence of ribonuclease A (RNase A) using the PNS of infected MDCK-F11A-CA cells (Figure 5D). If viral RNA polymerase (PB2, PB1, and PA) bound to the panhandle region of vRNA was the primary component, it should be coimmunoprecipitated with Rab11A even after RNase A treatment, but NP would be dissociated from the complex. Conversely, if NP was the primary target, NP but not viral RNA polymerase would be precipitated. If vRNA of vRNP itself was the primary component, both polymerase and NP would be sensitive to RNase A treatment. Our data show that coimmunoprecipitations of PB2, PB1, and PA with the CA mutant of FLAG-Rab11A were resistance to RNase A treatment and that of NP was apparently sensitive (Figure 5D, compare lane 1 and the others), suggesting that Rab11 interacts with vRNP through viral RNA polymerase, although viral/host factor(s)-mediated interaction cannot be ruled out.

### Overexpression of the Rab binding domains of Rab11 family interacting proteins inhibits localization of vRNP to RE

**Figure 6:**
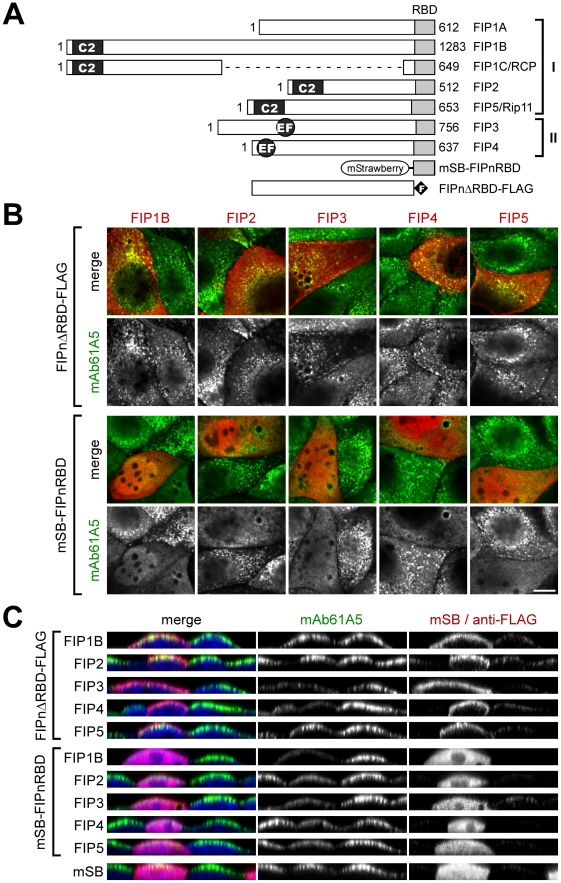
Effects of Rab11-FIP deletion mutants on the localization and trafficking of progeny vRNP segments. (**A**) Schematic representation of the functional domains of human Rab11-FIPs (FIPn). Numerals at both ends indicate amino acid residues. The Rab binding domains (RBD) of individual Rab11-FIPs were indicated as gray boxes. Typical Rab11-FIP1 gene products (FIP1A, -B, and -C/RCP) [96] were shown. The RBD fragment tagged with mStrawberry at the amino terminus (mSB-FIPnRBD) and the RBD deletion mutant containing a FLAG epitope tag at the carboxyl terminus (FIPnΔRBD-FLAG) were also illustrated. C2, C2-domain; EF, EF-hand domain. (**B**) Localization of progeny vRNPs in infected MDCK cells transiently expressing Rab11-FIP deletion mutants. Rab11-FIPs with deletion of RBD (upper two rows) and RBD fragments (lower two rows) were visualized using anti-FLAG mAb and mSB (red), respectively. Progeny vRNPs were also visualized using anti-NP mAb61A5 (green). Confocal merged images (odd rows) and vRNP-channel images (even rows) are shown. All images are shown at the same magnification. Scale bar  = 10 µm. (**C**) Polarized localization of progeny vRNP. XZ sections of polarized MDCK cells. Nuclei were stained with DAPI (blue) and shown in merged images (left images).

The Rab family protein is involved in a variety of cellular processes through interaction with specific effector proteins. In the case of Rab11, Rab11 family interacting proteins (Rab11-FIP1 to 5) have been identified as effector proteins (Figure 6A) [57], [58], [59], [60]. The Rab binding domains (RBDs), located at the carboxyl termini of Rab11-FIPs, are relatively conserved among Rab11-FIPs and interact with the switch regions of active form of Rab11 [61], [62], [63]. The other regions are involved in the effector functions of individual Rab11-FIPs [64], [65]. We examined if Rab11-FIP played an important role in the targeting of progeny vRNP to RE. We constructed RBD deletion (ΔRBD) mutants and RBD fragments of Rab11-FIPs and added a FLAG tag to the carboxyl termini of the ΔRBD mutants and monomeric red fluorescent protein, mStrawberry, to the amino termini of the RBD fragments (Figure 6A, FIPnΔRBD-FLAG and mSB-FIPnRBD, n = 1 to 5). Since Rab11-FIP ΔRBD mutants cannot bind with Rab11, overexpression of ΔRBD mutants might inhibit the effector functions of the corresponding endogenous Rab11-FIPs. However, none of Rab11-FIP ΔRBD mutants altered the localization of vRNP to RE (Figure 6B, FIPnΔRBD-FLAG). In contrast, all Rab11-FIP RBD fragments we tested impaired the localization of vRNP to RE (Figure 6B, mSB-FIPnRBD), implying that excess level of RBD expression might disrupt the Rab11-vRNP interaction.

### Apical transport of progeny vRNP depends on the endosomal recycling pathways

It is well known that influenza virus buds at the APM in polarized epithelial cells [23]. Our previous study indicated that vRNP signals were accumulated at the APM in polarized MDCK cells after 6 hpi [45]. Thus, we carefully observed the xz section images of infected MDCK cells. Consistent with the xy images (Figure 6B), marked accumulation of vRNP signals at the APM was not observed when Rab11-FIP RBD fragments were overexpressed (Figure 6C, mSB-FIPnRBD), suggesting that the APM accumulation of cytoplasmic vRNPs is not due to diffusion even though the apical side of nuclear membrane is close to the APM. When observed with Rab11-FIP ΔRBD mutants, we confirmed that class I Rab11-FIP ΔRBD mutants did not impair the APM accumulation of vRNPs (Figure 6C, Rab11-FIP1B/2/5ΔRBD). Interestingly, overexpression of class II Rab11-FIP ΔRBD mutants did not exhibit the APM accumulation of vRNPs (Figure 6C, Rab11-FIP3/4ΔRBD), although these mutants did not inhibit the targeting of vRNPs to RE (Figure 6B). It is plausible that overexpression of nonfunctional Rab11-FIP3/4 mutants disturbed the apical trafficking by disrupting the structural integrity of pericentriolar ERC/RE, as reported previously [66], [67]. Altogether, our data suggest that not only targeting of vRNP to RE but also functional apical recycling machinery are both required for membrane trafficking of progeny vRNPs and subsequent particle release.

## Discussion

In this study, we showed that (i) progeny vRNP of influenza A virus was localized at RE and transported along microtubules; (ii) The localization required the interaction between active/GTP-bound Rab11 and a heterotrimeric form of viral RNA-dependent RNA polymerase; and (iii) The Rab11-dependent interaction was required for the targeting of progeny vRNPs to the APM, where virion packaging and budding take place. Very recently, Amorim MJ *et al.* independently reported that cytoplasmic transport of influenza virus RNA genome required Rab11- and microtubule-dependent mechanisms [68]. Their conclusion is in good agreement with our result that genetically unmodified vRNPs moved along the microtubules in living cells. These independent studies confirm the usage of RE for influenza virus vRNP trafficking.

### Live cell imaging using fluorescent-labeled antibody transfection technique, and microtubule-dependent viral transport

For live cell imaging of vRNPs, we have transfected fluorescent-labeled mAb61A5 which preferentially recognized RNP complexes of influenza virus and have demonstrated that vRNP signals move along AcGFP-labeled microtubules rapidly but intermittently in both plus and minus directions (Figures 1 and 2, Videos S1 and S3). Thus, live cell imaging using fluorescent-labeled antibody may have advantages over a conventional technique of tagging with fluorescent protein (i) when the tagging impairs protein functions or trafficking and (ii) when the antibody specifically detects a certain population of protein of interest. This technique does not require special skills and equipments when compared with microinjection. The disadvantages of fluorescent-antibody transfection include the appearance of pseudo-positive signals, as shown in Figures 1 and 2. They are probably antibodies that were endocytosed non-specifically, or aggregated on the plasma membrane or with liposomes. Thus, cotransfection of non-specific control antibody and/or mock infection of inactivated virus are required to distinguish true signals from pseudo-positives. Another disadvantage would be a possible reduction in velocity because of large complex formation of antigen and antibody.

Previous studies indicated that HSV moved in axon of cultured nerve cell at 2–3 mm/h [69] and that organelles containing HSV capsids moved on *in vitro*-reconstituted microtubules at a mean velocity of 0.58 µm/s [70]. Sendai virus vRNP was visualized by tagging with fluorescent protein to L protein and velocity of the RE-dependent vRNP movement was calculated at subsecond temporal resolution (0.41–1.04 µm/s) [71]. These velocities were ostensibly comparable to the mean velocity of influenza virus progeny vRNPs observed in our study (Figure 1B, approximately 1.45 µm/s). Since cargos which are transported along microtubules by membrane vesicles moves rapidly but intermittently in both directions, subsecond temporal resolution must be required for accurate instantaneous velocity of their transport. A very recent report demonstrated that a fraction of reconstituted influenza virus vRNP showed saltatory movement at an average of 0.81 µm/s [68]. This mean velocity is slightly slower than but still comparable with our mean velocity of genetically unmodified vRNP in infected cells. Relatively lower temporal resolution (approximately every 4s), tagging with GFP, and/or the elapsed time from the transfection (24 hours posttransfection) may cause the velocity reduction observed in their study.

### Localization of progeny vRNP to RE via interaction between viral RNA polymerase and Rab11

Previous studies on transient coexpression of three subunits of viral RNA polymerase (PB2, PB1, and PA) have indicated that hetero-trimerization of the subunits takes place in the nucleus but not in the cytoplasm, showing a limited localization of heterotrimeric viral RNA polymerase in the nucleus [72], [73], [74]. In the infected cell, the heterotrimeric viral RNA polymerase is incorporated into progeny vRNP and then exported to the cytoplasm by CRM1-dependent nuclear export system [75], whereas cRNP which serves a template for vRNA synthesis remains in the nucleus [12]. These studies suggest that most of the heterotrimeric viral RNA polymerase in the cytoplasm exists as a constituent of progeny vRNP. In this study, our immunoprecipitation analysis with RNase A treatment revealed that active/GTP-bound Rab11 interacted with vRNP through the viral RNA polymerase but not NP or vRNA (Figure 5D), although it remains to be elucidated whether the interaction is direct or not. If a certain subunit or heterodimer could solely interact with Rab11, it would also be transported to the APM. However, it has been well known that not only singly expressed subunits but also coexpressed three subunits did not accumulate at the plasma membrane [73], [74]. Thus, we reasoned that the heterotrimeric viral RNA polymerase in vRNP might serve as a marker for RE-dependent apical transport of progeny vRNP, since Rab11, a resident of RE, binds to viral RNA polymerase. It has been suggested that enzymatic/structural state of viral RNA polymerase is probably altered by classes of associated RNAs, e.g., single-stranded RNA, panhandle region of vRNA, or that of cRNA [12], [76], [77]. The state of viral RNA polymerase may similarly serve as a marker for targeting of vRNP to RE and excluding of viral mRNP containing single-stranded viral mRNA, if present in the cytoplasm. We are currently investigating whether active/GTP-bound Rab11 directly interacts with a certain class of viral RNA polymerase, or another viral/host factor(s) is involved. Amorim MJ *et al.* have suggested that the Rab11-vRNP interaction is due to Rab11-PB2 subunit interaction [68]. In their study, coexpression of GFP-tagged CA Rab11 with PB2, PB1, PA, or NP and subsequent affinity precipitation of GFP-Rab11 resulted in the coprecipitation of PB2 but not the other viral components. Although it remains to be elucidated why PB2 subunit could solely interact with active/GTP-bound Rab11 in the cytoplasm, it is possible that PB2 in the heterotrimeric viral RNA polymerase complex in a certain enzymatic/structural state participates in the Rab11-vRNP interaction.

### A model for a higher-order assembly of progeny vRNP segments on a Rab11-positive membrane

Recent studies have suggested that viral membrane/matrix proteins of some viruses traffic via endosomal pathways [13], [14]. However, the intracellular trafficking of viral inner components has long been less understood. In this study, we identified RE as a target compartment of influenza virus progeny vRNP. A possible explanation for the utilization of RE is that the surface of RE is a place for a higher-order assembly of vRNP segments (for review, see [78]). From studies with defective-interfering viral RNAs [79], [80], [81], it has been widely accepted that eight distinct segments of progeny vRNP are selectively packaged into a virion. Recent reports have shown that the approximately 150 to 200 base sequences at both termini of vRNA segments are responsible for their selective packaging into virions [82], [83], although it has not been demonstrated whether the putative inter-vRNP base pairing through the terminal regions is the molecular basis of the selective assembly and/or packaging. If it was the case, intracellular localization, local concentration, and spatial orientation of the terminal regions would be of great importance.

**Figure 7:**
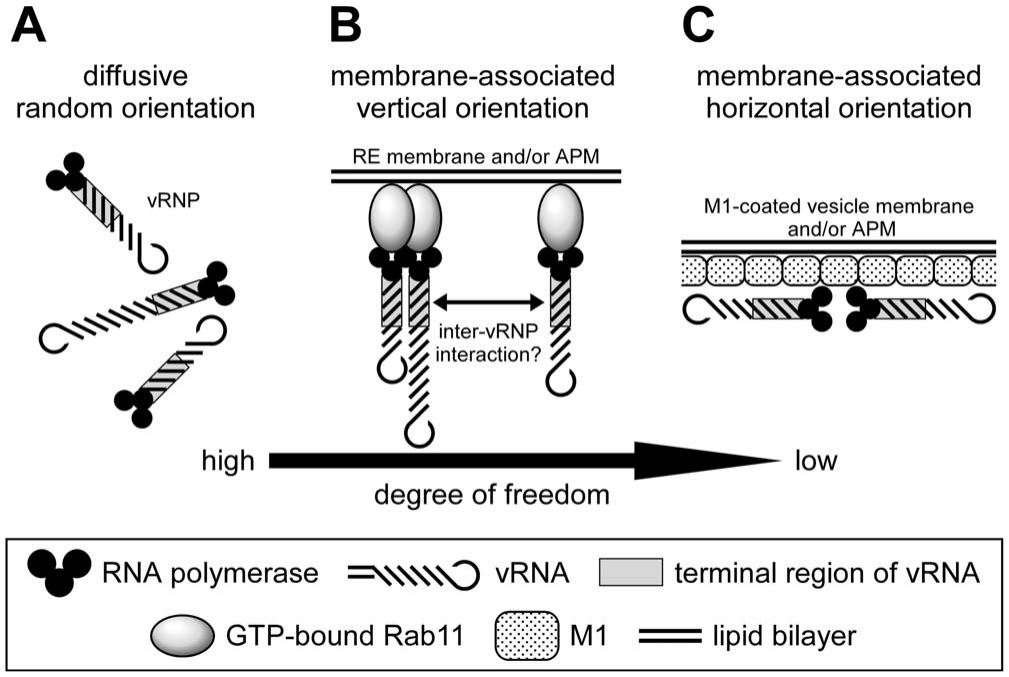
Models for spatial orientation of vRNP segments toward a higher-order assembly. Putative spatial orientations of progeny vRNP segments in the cytoplasm were illustrated. (**A**) Diffusive random orientation model, (**B**) membrane-associated vertical orientation model may occur on RE and/or beneath the APM, and (**C**) membrane-associated horizontal orientation model may occur on a vesicle and/or beneath the APM precoated with M1. Details were described in the Discussion section.

We propose the models for a higher-order assembly of vRNP segments (Figure 7). The most likely scenario (Figure 7B) would be that (i) the progeny vRNP segments bind to RE membrane (Figure 3, panels A and E) through interaction of active/GTP-bound Rab11 and heterotrimeric viral RNA polymerase (Figure 5D), followed by trafficking to the APM along microtubules (Figure 2). (ii) Because viral RNA polymerase is associated with the panhandle region of vRNA where is close to the sequences necessary for genome packaging (gray box), these terminal regions are concentrated and aligned in the same orientation on the RE membrane and later at the APM. (iii) By lateral diffusion, each vRNP segment slides on the membrane surface relatively freely to seek the others. This mild spatial restriction may allow a higher-order assembly of vRNP segments in a “try and select” manner, leading to packaging of eight vRNP segments into a virion, as observed by electron microscopy [84], [85].

If vRNP segments were freely diffusible in the cytosol (Figure 7A), the frequency of putative inter-vRNP interaction in a correct orientation would be very low. In fact, coexpressed with the DN mutant of Rab11 and Rab11-FIP RBD fragments (Figure 4A and 6B, respectively), vRNPs remained diffuse and were not seen as puncta, suggestive of a failure of a higher-order assembly of vRNP segments. Consistently, the production of infectious virions from the cells expressing the DN mutant was markedly decreased (Figure 4B), although perturbation of Rab11-dependent budding events cannot be ruled out [38]. An alternative model would be assembly of vRNP segments on M1-precoated vesicle/membrane (Figure 7C) as suggested previously (for review, see [86]). If NP and/or vRNA in a vRNP interacted directly with M1 [87], [88], [89], vRNP segments would be immobilized on the M1-coated membrane and fail to assemble each other. Recent electron microscopic analysis has suggested no such a tight association of vRNP with the electron-dense M1 layer in virions [84], [85]. Neither progeny vRNP signals detected by mAb61A5 nor by FISH analysis colocalized with HA/M1 antigens in the cytoplasm (Figure S2 and [45]). These results suggest that progeny vRNP and HA/M1 are transported independently through distinct apical transport pathways [90].

### Rab11, a key player in trafficking of non-membrane-bound cytoplasmic viral/cellular factors

In the past three decades, endosomal recycling has been extensively investigated. The majority of cargos analyzed are membrane-bound proteins/complexes and membrane lipids, e.g., transferrin-transferrin receptor complexes and endocytic transport to the cleavage furrow during cytokinesis. The well-known non-membrane-bound cytoplasmic cargos of RE are Rab11 effectors and motor proteins. Recent virological studies suggest independently the utilization of RE for viral trafficking and egress: cytoplasmic transport of hantavirus [91], apical budding of RSV [35], [92], cytoplasmic envelopment of human cytomegalovirus [93], and budding of influenza A virus [38]. It has been reported that the RE machinery is also used for vRNP trafficking of Sendai virus [71] and most recently for the trafficking of the influenza virus RNA genome [68], independently of our study. In this study, we reported that Rab11 recognized a non-membrane-bound molecule, i.e., progeny vRNP, and transported from the perinuclear region to the APM via RE. Collectively, these data strongly suggest that the utilization of Rab11-driven endosomal recycling system is a common transport mechanism of viral and possibly cellular non-membrane-bound cytoplasmic cargos. Budding of influenza A virus has been shown to occur independently of the ESCRT machinery [36], [37] but to require the Rab11-mediated machinery [38], suggesting that influenza virus may require a Rab11-related molecule(s) for virion release. It is tempting to speculate that vRNP segments and a factor(s) necessary for virion budding/pinching-off meet on a Rab11-positive RE and are transported together to the APM. Viral M2 protein is a candidate of such a factor since it has been reported that M2 protein mediates ESCRT-independent membrane scission and knock-down of Rab11 leads to a statistically significant reduction in the levels of M2 from the cell surface [94].

Our present study provides an outline of intracellular trafficking of influenza viral replication complex, vRNP, from the nucleus, a site of viral genome replication, to the APM, a site of genome packaging and virion budding. However, many elementary steps of the trafficking remain to be elucidated. For examples, an intracellular site where progeny vRNPs initially ride on Rab11-positive RE and motor proteins involved in the apical trafficking of vRNPs need to be identified. Investigation of these elementary steps will reveal precise molecular mechanisms of apical trafficking and a higher-order assembly of progeny vRNP segments for genome packaging. Our study may also provide a clue to the transport mechanisms of host cellular non-membrane-bound cytoplasmic cargos such as mRNP trafficking followed by local protein translation.

## Materials and Methods

Materials and Methods for antibodies, DNA construction, establishment of cell lines, and immunofluorescent microscopy were described in Materials and Methods S1. Oligonucleotide sequences used for DNA construction were described in Tables S2, S3, S4 and S5.

### Live cell imaging

MDCK cells were cultured in Dulbecco's modified Eagle medium (DMEM, Cat. No. D5796, Sigma-Aldrich, USA) containing 10% fetal bovine serum (FBS) on φ35 mm glass-bottom dishes and infected with influenza virus A/Puerto Rico/8/34 (PR8) strain at moi of 3 for 1 h. Residual viral inoculum was digested with 80 µg/ml of acetyl-trypsin in serum-free medium (Opti-MEM I, Life Technologies, USA) for 2 h and followed by masking with 0.2 mg/ml of unlabeled mAb61A5 for 30 min. At 3.5 hpi, 400 ng of Alexa Fluor 568 (AF568)-labeled mAb61A5 was transfected together with 400 ng of AF488-labeled non-specific mouse immunoglobulin (control antibody), using protein transfection reagent (Ab-DeliverIN, OZ Biosciences, France) according to the manufacturer's instruction. At 7 hpi, the medium was exchanged to DMEM for live cell imaging (Cat. No. 21063-029, Life Technologies) containing 10% FBS.

Live cell imaging was performed using a fluorescence microscope (IX71, Olympus Optical, Japan) equipped with an oil immersion objective lens (Plan Apo N, 60x, 1.42NA, Olympus), a stage top incubation chamber (Tokai HIT, Japan), a microlens-enhanced Nipkow-disk confocal scanner unit (CSU-X1, Yokogawa Electric, Japan), an optical filter wheel controller, and an electron multiplying CCD camera (Luca, Andor Technology, UK). For pseudo-positive signals, both fluorescent images with mAb61A5 and control antibody were acquired alternately (0.25 to 0.50 s exposure/image) with Ar laser excitation (488 or 568 nm) and were merged (Figure 1A, merge; Video S1, the first color part). Immediately after the dual-color acquisition, single channel acquisition of mAb61A5 images was carried out at 0.25 s exposure/image (Video S1, the second gray scale part). Sequential images were processed by using ImageJ software [95] as follows: (i) bleach correction, (ii) subtraction of a time projection image of mean intensity from fluorescence images at each time point, (iii) contrast correction (Video S1, the third part), and (iv) tracking of punctate fluorescent signals by using MTrackJ plugin created by Eric Meijering (http://www.imagescience.org/meijering/software/mtrackj/) (Video S1, the fourth part with trajectories).

### Kinetic analysis of fluorescent signals

Coordinates of vRNP signals at each time point were obtained from trajectories. An instantaneous velocity (v_n_, n means a frame number) and a vector (***V***_n_) of a signal at each time point were calculated from coordinates n (x_n_, y_n_), n+1 (x_n+1_, y_n+1_), and a frame interval (0.25 s). One motile event was defined as a single unidirectional movement of a signal, when the movement from a start point (frame number s) to an end point (frame number e) fulfills the following conditions: (i) v_n_ >0.13 µm/s (n is s to e-1), (ii) a relative angle between vectors ***V***_n_ and ***V***_n+1_ <±60° (n is s to e-2), and (iii) at least four sequential time points, i.e., a duration is no fewer than 0.75 s when frame interval is 0.25 s. The threshold velocity (0.13 µm/s) was determined by the mean of instantaneous velocities of vRNP signals in pausing conditions. The angle threshold (±60°) was estimated from the maximum curvature of microtubules observed by immunofluorescence microscopy and the maximum velocity 10 µm/s we tentatively assigned. Mean and maximum of instantaneous velocities (V_mean_ and V_max_, respectively) and migration length of one motile event were calculated (Table S7) and plotted as histograms.

### Immunoprecipitation

MDCK-Neo, MDCK-F11A-WT, -DN, and -CA cells were seeded into φ10 cm dishes (3×10^6^ cells/dish). After incubation for 12 h, cells were infected with influenza virus PR8 strain at moi of 1 for 1 h. At 7 hpi, cells were harvested with 1 ml of cold PBS containing 0.1% Tween-20 (PBS-T), 1 mM DTT, 0.1 mM GTPγS (JENA Bioscience, Germany), 100 ng/µl of BSA, 0.5 U/µl of RNase inhibitor (Toyobo, Japan), and protease inhibitor cocktail (Cat. No. 25955-11, Nacalai Tesque, Japan). Cells were passed through 26G needle 20 strokes and the PNS was isolated by centrifugation at 4°C, at 1,000× g for 10 min. One milliliter of PNS was mixed with 20 µg of anti-FLAG mAb and incubated on ice for 1 h. The PNS was subsequently mixed with pre-blocked 20 µl packed-volume (p.v.) of Protein G Mag Sepharose (GE Healthcare, UK) and rotated at 4°C for 2 h. After being washed twice with PBS-T, immunoprecipitates were eluted twice with 50 µl of PBS-T containing 150 ng/µl of 3×FLAG peptide (Sigma-Aldrich) for 30 min (total 60 min and 100 µl of eluate). The eluate and PNS were analyzed by Western blotting. Similarly, immunoprecipitation of viral RNP complexes were carried out with 20 µg of anti-NP mAb61A5 and eluted twice with 25 µl of PBS-T containing 100 ng/µl RNase A at 25°C for 30 min (total 60 min and 50 µl of eluate).

 For RNase sensitivity assay, PNS of infected MDCK-F11A-CA cells were similarly prepared except for RNase inhibitor. Following addition of anti-FLAG mAb to the PNS, 250 µl of aliquots were incubated with 0 to 100 ng/µl of RNase A at 25°C for 1 h and precipitated by using 5 µl p.v./assay of Protein G Mag Sepharose at 25°C for 2 h. Elution was carried out twice with 25 µl of elution buffer at 25°C for 30 min (total 60 min and 50 µl of eluate).

### Reverse transcription and semiquantitative real-time PCR

RNA was isolated from immunoprecipitation eluate (Fig. 5C) and PNS (Figure S3) by using RNeasy mini kit (Qiagen, Germany). Equal volume of each RNA sample was used for reverse transcription (ReverTra Ace qPCR RT Kit, Toyobo) in the presence of a primer mixture containing 2 pmol each of eight segment-specific primers, which is either for negative- (vRNA) or positive-sense (c/mRNA) influenza virus RNAs (Table S6). Semiquantitative real-time PCR (qPCR) was carried out (SYBR Premix Ex Taq II and Real Time PCR System TP800, Takara Bio, Japan) in the presence of each segment-specific qPCR primer pair (reverse transcription product ×8 qPCR reactions). Threshold cycles (Ct) were obtained by second derivative maximum method.

For the standard DNA of segment-specific qPCR, short cDNA fragments to individual viral RNA segments were amplified using qPCR primer pairs, concatenated, and cloned into pBluescript-SK(+) (Agilent Technologies, USA) (see Materials and Methods S1 and Figure S1B). The resultant plasmid (pBSPR8qPCRSTD) has one copy each of eight qPCR target sequences. Standard curves for Ct values of individual targets vs. cDNA concentrations were obtained using ten-fold dilutions of this standard DNA (0.0001 to 0.1 fmol/reaction) and were used for relative quantification of reverse-transcribed cDNA segments.

## Supporting Information

Figure S1 **DNA construction of expression vectors and the standard DNA plasmid for qPCR.** (A) DNA sequences of pCANeoHA and pCANeoAcGFP-MCS. The DNA sequences corresponding to the region between two *Eco*R I sites of original pCAGGS were shown. The positions of cloning sites, HA epitope tag, and AcGFP tag were indicated. Amino acid sequences were also shown. (B) Construction scheme of the qPCR standard plasmid (pBSPR8qPCRSTD) containing one copy each of eight distinct target sequences. Numerals, segment numbers of the influenza virus genome; white circles, 5′-phosphorylated. Details were described in Materials and Methods S1.
(TIF)

Figure S2 **Localizations of progeny vRNP and hemagglutinin in the cytoplasm.** MDCK cells were infected with PR8 strain for 1 h and 20 μM of brefeldin A (BFA), a vesicular transport inhibitor, was added at 4 h postinfection (hpi). Following fixation at 7 hpi, immunofluorescence staining was carried out as follows: (i) staining with anti-HA mAb and Alexa Fluor 488 dye (AF488)-conjugated anti-mouse Ig, (ii) post-fixation with 4% paraformaldehyde and blocking with non-specific mouse Ig, and (iii) staining with AF568-conjugated mAb61A5. Cells were observed with a confocal laser scanning microscope. Areas in white boxes were enlarged. In the presence of brefeldin A, membrane transport of HA was partially inhibited and a fraction of HA accumulated at the perinuclear region. An arrowhead shows a filamentous vRNP signal observed in the presence of BFA. Bars are 20 μm and 5 μm, respectively.
(TIF)

Figure S3 **Molar ratios of viral negative/positive-sense RNA segments in PNSs of infected MDCK-F11A-DN/CA cells.** Total RNAs were purified from infected cells and polarity-specific reverse transcription followed by segment-specific semiquantitative real-time PCR was carried out. Amounts of the cDNAs reverse-transcribed from viral RNAs were quantified using standard plasmid DNA containing single copy of each target sequence (pBSPR8qPCRSTD). Segment numbers were indicated at the bottom. Columns indicated the molar ratio of vRNAs (gray and black columns) and c/mRNAs (hatched and white columns) from MDCK-F11A-DN and -CA, when the segment 1 vRNA from MDCK-F11A-DN was set at 1.0.
(TIF)

Table S1 **List of Analyzed Rab Family Proteins and Their Cloning Information.**
(DOC)

Table S2 **Oligonucleotide Sequences. Used for the Cloning of Rab Family Proteins.**
(DOC)

Table S3 **Oligonucleotide Sequences Used for the Construction of Dominant Negative and Constitutively Active Mutants of Human Rab11A.**
(DOC)

Table S4 **Oligonucleotide Sequences Used for AcGFP- or FLAG-tagged Rab Family Protein Expression Vectors.**
(DOC)

Table S5 **Oligonucleotide Sequences Used for the Construction of Rab11-FIPs Deletion Mutant Expression Vectors.**
(DOC)

Table S6 **Oligonucleotide Sequences Used for Polarity-specific Reverse Transcription and Segment-specific Semiquantitative PCR.**
(DOC)

Table S7 **Mean and Maximum Velocities and Migration Lengths of Individual Motile Events.**
(DOC)

Video S1 **A representative live cell imaging and tracking of cytoplasmic progeny vRNP signals.** Live cell imaging of infected MDCK cells (Figure 1A) was carried out as described in the Materials and Methods section. Acquired images were processed, analyzed, and encoded to a movie containing concatenated four parts. The first color part contains 25 of merged images (red, mAb61A5 channel; green, control antibody channel). Each of single channel images was acquired alternately at 250 ms exposure. The second gray-scale part contains 100 images acquired at 250 ms exposure for 25 seconds at single mAb61A5 channel, immediately after the dual-color acquisition. The third part of the movie is post-processing images of the second part. The image processing procedure was described in the Materials and Methods section. The last part is the signal tracking by using ImageJ software and MTrackJ plugin (created by Eric Meijering, http://www.imagescience.org/meijering/software/mtrackj/). Individual signals were tracked manually (90 tracks). Track numbers, trajectories, and current position of vRNP signals were indicated on the post-processing images with numerals, colored lines, and blank circles, respectively. Elapsed times were also indicated.
(MPG)

Video S2 **Live cell imaging of mock-infected MDCK cells.** MDCK cells were infected with influenza A virus PR8 strain (right half) or mock-infected with heat-inactivated virus (left half). For live cell imaging, each of single channel images (red, mAb61A5 channel; green, control antibody channel) was acquired alternately at 500 ms exposure/image for 24 seconds and then merged. Bleach correction and contrast correction were carried out.
(MPG)

Video S3 **Live cell imaging of infected MDCK cells expressing AcGFP-α-tubulin.** MDCK-Tub cells, constitutively expressing AcGFP-α-tubulin, were infected with influenza A virus PR8 strain (Figure 2). For live cell imaging, each of single channel images (red, mAb61A5 channel; green, control antibody and AcGFP channel) was acquired alternately at 300 ms exposure/image for 60 seconds and then merged. Bleach correction and contrast correction were carried out. Cropped area (shown in Figure 2B) was encoded as a movie containing concatenated merged images, mAb61A5 channel images, and control antibody/AcGFP channel images. Elapsed times were indicated.
(MPG)

Materials and Methods S1 Details of the antibodies utilized in this study and methods for DNA construction, establishment of cell lines, and immunofluorescent microscopy were described.
(DOC)
