# Supplementary figures and images for: Apical Transport of Influenza A Virus Ribonucleoprotein Requires Rab11-positive Recycling Endosome
Source: PLoS One. 2011 Jun 22;6(6):e21123. doi: 10.1371/journal.pone.0021123 (PMC3120830; doi:10.1371/journal.pone.0021123)

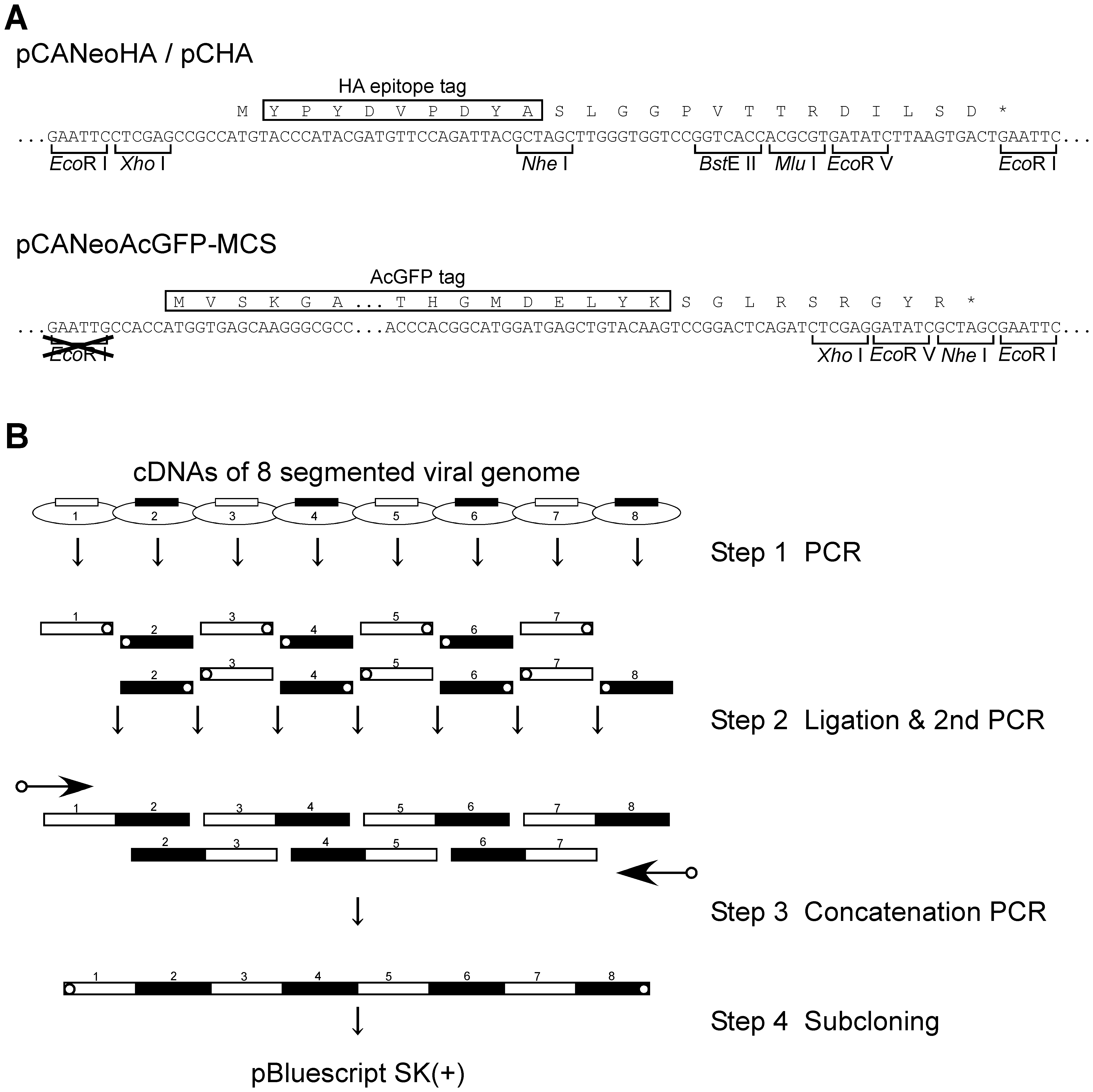

Supplement: Figure S1 — DNA construction of expression vectors and the standard DNA plasmid for qPCR. (A) DNA sequences of pCANeoHA and pCANeoAcGFP-MCS. The DNA sequences corresponding to the region between two EcoR I sites of original pCAGGS were shown. The positions of cloning sites, HA epitope tag, and AcGFP tag were indicated. Amino acid sequences were also shown. (B) Construction scheme of the qPCR standard plasmid (pBSPR8qPCRSTD) containing one copy each of eight distinct target sequences. Numerals, segment numbers of the influenza virus genome; white circles, 5′-phosphorylated. Details were described in Materials and Methods S1. (TIF) [file pone.0021123.s001.tif]

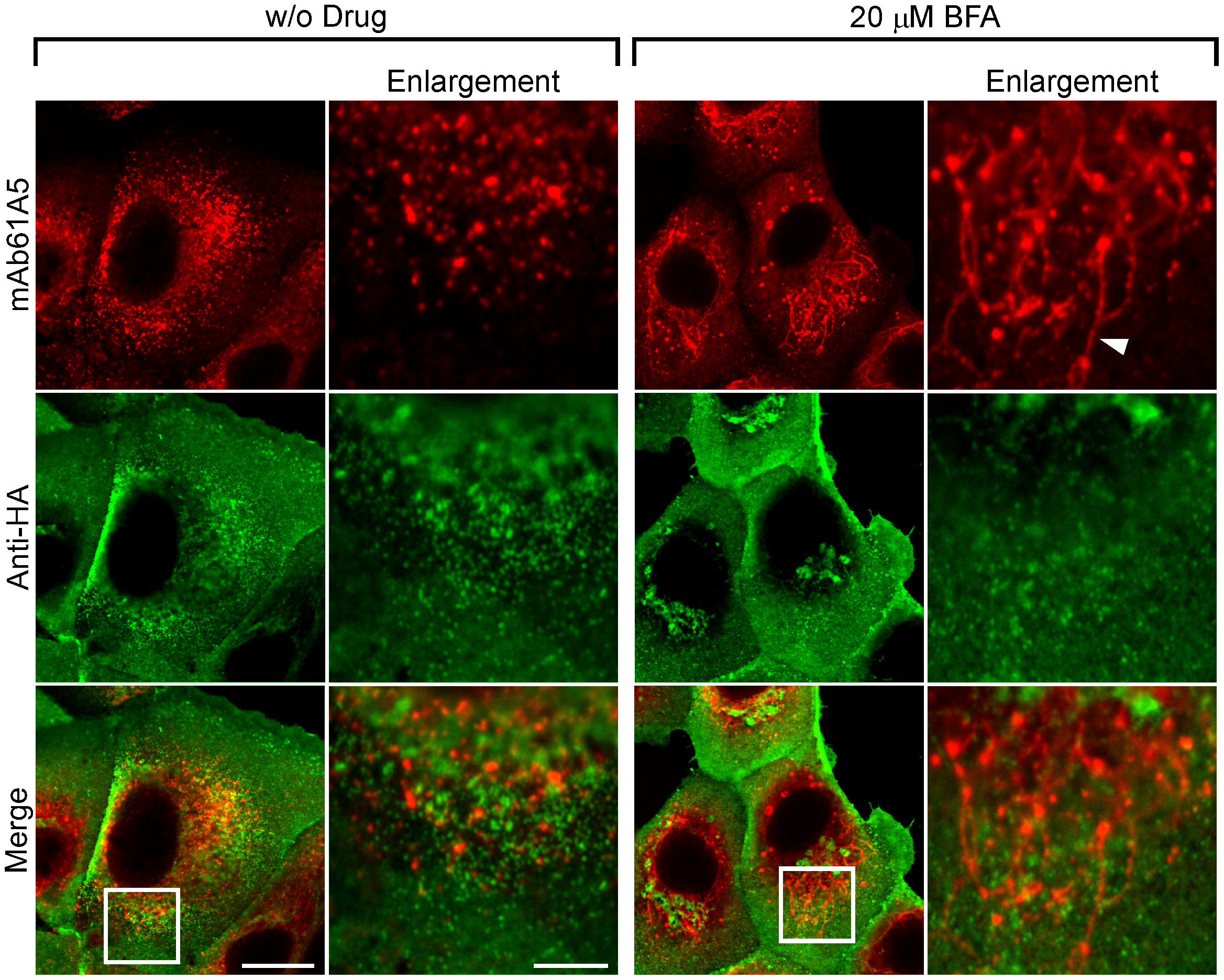

Supplement: Figure S2 — Localizations of progeny vRNP and hemagglutinin in the cytoplasm. MDCK cells were infected with PR8 strain for 1 h and 20 μM of brefeldin A (BFA), a vesicular transport inhibitor, was added at 4 h postinfection (hpi). Following fixation at 7 hpi, immunofluorescence staining was carried out as follows: (i) staining with anti-HA mAb and Alexa Fluor 488 dye (AF488)-conjugated anti-mouse Ig, (ii) post-fixation with 4% paraformaldehyde and blocking with non-specific mouse Ig, and (iii) staining with AF568-conjugated mAb61A5. Cells were observed with a confocal laser scanning microscope. Areas in white boxes were enlarged. In the presence of brefeldin A, membrane transport of HA was partially inhibited and a fraction of HA accumulated at the perinuclear region. An arrowhead shows a filamentous vRNP signal observed in the presence of BFA. Bars are 20 μm and 5 μm, respectively. (TIF) [file pone.0021123.s002.tif]

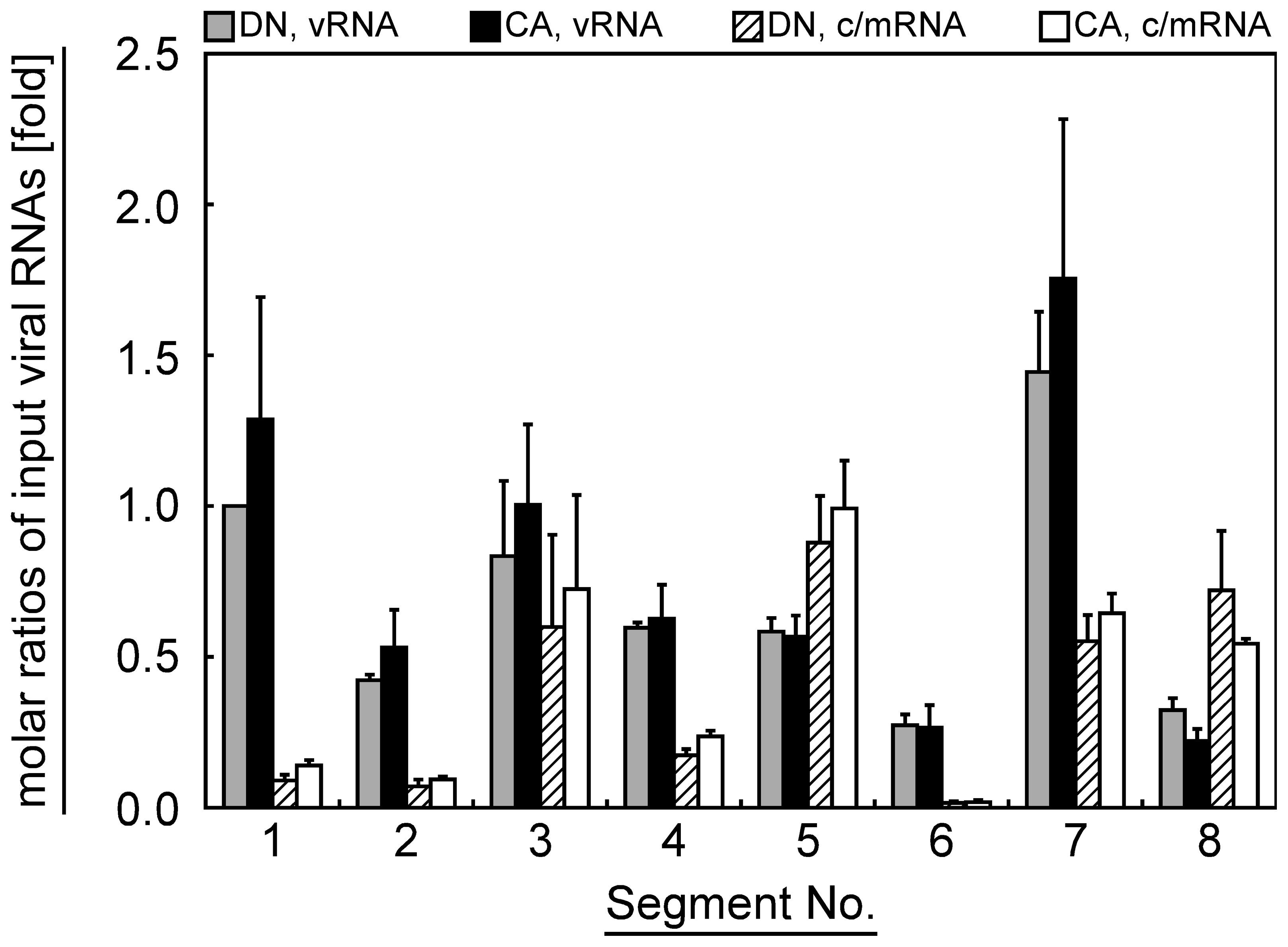

Supplement: Figure S3 — Molar ratios of viral negative/positive-sense RNA segments in PNSs of infected MDCK-F11A-DN/CA cells. Total RNAs were purified from infected cells and polarity-specific reverse transcription followed by segment-specific semiquantitative real-time PCR was carried out. Amounts of the cDNAs reverse-transcribed from viral RNAs were quantified using standard plasmid DNA containing single copy of each target sequence (pBSPR8qPCRSTD). Segment numbers were indicated at the bottom. Columns indicated the molar ratio of vRNAs (gray and black columns) and c/mRNAs (hatched and white columns) from MDCK-F11A-DN and -CA, when the segment 1 vRNA from MDCK-F11A-DN was set at 1.0. (TIF) [file pone.0021123.s003.tif]
