# Supplementary material for: Apical Transport of Influenza A Virus Ribonucleoprotein Requires Rab11-positive Recycling Endosome
Source: PLoS One. 2011 Jun 22;6(6):e21123. doi: 10.1371/journal.pone.0021123 (PMC3120830; doi:10.1371/journal.pone.0021123)
Supplement: Table S1 — List of Analyzed Rab Family Proteins and Their Cloning Information. (DOC) [file pone.0021123.s004.doc]

# Table S1

List of Analyzed Rab Family Proteins and Their Cloning Information.

| **Gene ID ^a^** | **Gene symbol ^b^** | **Cloning sites** | **Forward primer name ^c^** | **Reverse primer name** |
| --- | --- | --- | --- | --- |
| 5861 | RAB1A | *Xho* I, *Nhe* I | XhoNco-hRab1A(S2A)-F | hRab1A-StpNhe-Rev |
| 5862 | RAB2A | *Xho* I, *Nhe* I | XhoNco-hRab2A-For | hRab2A-StpNhe-Rev |
| 5867 | RAB4A | *Xho* I, *Eco*R I | XhoNco-hRab4A(S2A)-F | hRab4A-StpER1-Rev |
| 5869 | RAB5B | *Xho* I, *Eco*R I | XhoNco-hRab5B(T2A)-F | hRab5B-StpER1-Rev |
| 5870 | RAB6A | *Xho* I, *Nhe* I | XhoNco-hRab6A(S2A)-F | hRab6A-StpNhe-Rev |
| 7879 | RAB7A | *Xho* I, *Nhe* I | XhoNco-hRab7A(T2A)-F | hRab7A-StpNhe-Rev |
| 4218 | RAB8A | *Xho* I, *Bam*H I | XhoNco-hRab8a-For | hRab8a-Eco1Bam-Rev |
| 9367 | RAB9A | *Xho* I, *Nhe* I | XhoNco-hRab9A-For | hRab9A-StpNhe-Rev |
| 10890 | RAB10 | *Xho* I, *Nhe* I | XhoNco-hRab10-For | hRab10-StpNhe-Rev |
| 8766 | RAB11A | *Xho* I, *Bam*H I | XhoNco-hRab11a-For | hRab11a-Eco1Bam-Rev |
| 9230 | RAB11B | *Xho* I, *Bam*H I | XhoNco-hRab11b-For | hRab11b-Eco1Bam-Rev |
| 51552 | RAB14 | *Xho* I, *Nhe* I | XhoNco-hRab14-For | hRab14-StpNhe-Rev |
| 376267 | RAB15 | *Xho* I, *Nhe* I | XhoNco-hRab15-For | hRab15-StpNhe-Rev |
| 64287 | RAB17 | *Xho* I, *Nhe* I | XhoNco-hRab17-For | hRab17-StpNhe-Rev |
| 57403 | RAB22A | *Xho* I, *Nhe* I | XhoNco-hRab22A-For | hRab22A-StpNhe-Rev |
| 51715 | RAB23 | *Xho* I, *Nhe* I | XhoNco-hRab23(L2V)-F | hRab23-StpNhe-Rev |
| 57111 | RAB25 | *Xho* I, *Eco*R I | XhoNco-hRab25-For | hRab25-StpER1-Rev |
| 11031 | RAB31 | *Xho* I, *Nhe* I | XhoNco-hRab31(M2V)-F | hRab31-StpNhe-Rev |
| 83452 | RAB33B | *Eco*R V, *Eco*R I | Nco-hRab33B-For | hRab33B-StpER1-Rev |
| 83871 | RAB34 | *Xho* I, *Nhe* I | XhoNco-hRab34(N2A)-F | hRab34-StpNhe-Rev |
| 11021 | RAB35 | *Xho* I, *Nhe* I | XhoNco-hRab35-For | hRab35-StpNhe-Rev |

^a^ NCBI gene ID (http://www.ncbi.nlm.nih.gov/gene).

^b^ These Rab family proteins were selected based on the review [Schwartz SL, et al., (2007) Rab GTPases at a glance. J Cell Sci 120: 3905-3910].

^c^ Point substitutions were indicated in brackets.
