# Supplementary material for: Apical Transport of Influenza A Virus Ribonucleoprotein Requires Rab11-positive Recycling Endosome
Source: PLoS One. 2011 Jun 22;6(6):e21123. doi: 10.1371/journal.pone.0021123 (PMC3120830; doi:10.1371/journal.pone.0021123)
Supplement: Table S2 — Oligonucleotide Sequences. Used for the Cloning of Rab Family Proteins. (DOC) [file pone.0021123.s005.doc]

# Table S2

Oligonucleotide Sequences Used for the Cloning of Rab Family Proteins.

| **Primer name ^a^** | **Sequence (5' to 3')** |
| --- | --- |
| XhoNco-hRab1A(S2A)-F | GACTCGAGCCATGGCCAGCATGAATCCCGAATA |
| hRab1A-StpNhe-Rev | GTAGCTAGCTTAGCAGCAACCTCCACCTGACT |
| XhoNco-hRab2A-For | GACTCGAGCCATGGCGTACGCCTATCTCTTCAA |
| hRab2A-StpNhe-Rev | GTAGCTAGCTCAACAGCAGCCGCCCCCAGCCT |
| XhoNco-hRab4A(S2A)-F | GACTCGAGCCATGGCGCAGACGGCCATGTCCGAAA |
| hRab4A-StpER1-Rev | GTAGAATTCCTAACAACCACACTCCTGAGCGTTCG |
| XhoNco-hRab5B(T2A)-F | GACTCGAGCCATGGCTAGCAGAAGCACAGCTAGG |
| hRab5B-StpER1-Rev | GTAGAATTCTCAGTTGCTACAACACTGGCTCTT |
| XhoNco-hRab6A(S2A)-F | GACTCGAGCCATGGCCACGGGCGGAGACTTCGGGA |
| hRab6A-StpNhe-Rev | GTAGCTAGCTTAGCAGGAACAGCCTCCTTCACT |
| XhoNco-hRab7A(T2A)-F | GACTCGAGCCATGGCCTCTAGGAAGAAAGTGTTGC |
| hRab7A-StpNhe-Rev | GTAGCTAGCTCAGCAACTGCAGCTTTCTGCCGAGG |
| XhoNco-hRab8a-For | GCCTCGAGCCATGGCGAAGACCTACGATTACCTGT |
| hRab8a-Eco1Bam-Rev | CCGGATCCGAATTCACAGAAGAACACATCGGAAAAAG |
| XhoNco-hRab9A-For | GACTCGAGCCATGGCAGGAAAATCATCACTTTTTA |
| hRab9A-StpNhe-Rev | GTAGCTAGCTCAACAGCAAGATGAGCTAGGCTTGG |
| XhoNco-hRab10-For | GACTCGAGCCATGGCGAAGAAGACGTACGACCTGC |
| hRab10-StpNhe-Rev | GTAGCTAGCTCAGCAGCATTTGCTCTTCCAGCCT |
| XhoNco-hRab11a-For | GCCTCGAGCCATGGGCACCCGCGACGACGAGTA |
| hRab11a-Eco1Bam-Rev | CCGGATCCGAATTCTTAGATGTTCTGACAGCACTGCACC |
| XhoNco-hRab11b-For | GCCTCGAGCCATGGGGACCCGGGACGACGAGTA |
| hRab11b-Eco1Bam-Rev | CCGGATCCGAATTCTCACAGGTTCTGGCAGCACTGCAGC |
| XhoNco-hRab14-For | GACTCGAGCCATGGCAACTGCACCATACAACTACT |
| hRab14-StpNhe-Rev | GTAGCTAGCCTAGCAGCCACAGCCTTCTCTCTGGG |
| XhoNco-hRab15-For | GACTCGAGCCATGGCGAAGCAGTACGATGTGCTGT |
| hRab15-StpNhe-Rev | GTAGCTAGCTCAGCACCAGCAGGTTTTCGAAGAGT |
| XhoNco-hRab17-For | GACTCGAGCCATGGCACAGGCACACAGGACC |
| hRab17-StpNhe-Rev | GTAGCTAGCCTAGTGGGCGCAGCATTTGGCCT |
| XhoNco-hRab22A-For | GACTCGAGCCATGGCGCTGAGGGAGCTCAAAGTGT |
| hRab22A-StpNhe-Rev | GTAGCTAGCTCAGCAGCAGCTCCGCTTTGGCTCT |
| XhoNco-hRab23(L2V)-F | GACTCGAGCCATGGTGGAGGAAGATATGGAAGTC |
| hRab23-StpNhe-Rev | GTAGCTAGCTTAGGGTATGCTACAGCTGCTAAAA |
| XhoNco-hRab25-For | GACTCGAGCCATGGGGAATGGAACTGAGGAAGAT |
| hRab25-StpER1-Rev | GTAGAATTCTCAGAGGCTGATGCAACAGGCCC |
| XhoNco-hRab31(M2V)-F | GACTCGAGCCATGGTGGCGATACGGGAGCTCAAAG |
| hRab31-StpNhe-Rev | GTAGCTAGCTCAACAGCACCGGCGGCTGGCTT |
| Nco-hRab33B-For | CCATGGCTGAGGAGATGGAGTCGTCGCTCGAGGCA |
| hRab33B-StpER1-Rev | GTAGAATTCTTAGCACCAGCACGTCATTGCAGGC |
| XhoNco-hRab34(N2A)-F | GACTCGAGCCATGGCCATTCTGGCACCCGTGCGGA |
| hRab34-StpNhe-Rev | GTAGCTAGCTCATGGGCAACATGTGGGCTTCTTCT |
| XhoNco-hRab35-For | GACTCGAGCCATGGCCCGGGACTACGACCACCTCT |
| hRab35-StpNhe-Rev | GTAGCTAGCTTAGCAGCAGCGTTTCTTTCGTTTAC |

^a^ Point substitutions were indicated in brackets.
