# Supplementary material for: Apical Transport of Influenza A Virus Ribonucleoprotein Requires Rab11-positive Recycling Endosome
Source: PLoS One. 2011 Jun 22;6(6):e21123. doi: 10.1371/journal.pone.0021123 (PMC3120830; doi:10.1371/journal.pone.0021123)
Supplement: Table S3 — Oligonucleotide Sequences Used for the Construction of Dominant Negative and Constitutively Active Mutants of Human Rab11A. (DOC) [file pone.0021123.s006.doc]

# Table S3

Oligonucleotide Sequences Used for the Construction of Dominant Negative and Constitutively Active Mutants of Human Rab11A.

| **Primer name** | **Sequence (5' to 3')** |
| --- | --- |
| hRab11A-S25NNLL-For | AATAATCTCCTGTCTCGATTTACTCGAA |
| hRab11A-GVGK24-Rev | CTTTCCAACACCAGAATCTCCAATAAG |
| hRab11A-Q70LERY-For | CTAGAGCGATATCGAGCTATAACATCAGCA |
| hRab11A-DTAG69-Rev | CCCTGCTGTGTCCCATATCTGTG |
