# Supplementary material for: Apical Transport of Influenza A Virus Ribonucleoprotein Requires Rab11-positive Recycling Endosome
Source: PLoS One. 2011 Jun 22;6(6):e21123. doi: 10.1371/journal.pone.0021123 (PMC3120830; doi:10.1371/journal.pone.0021123)
Supplement: Table S4 — Oligonucleotide Sequences Used for AcGFP- or FLAG-tagged Rab Family Protein Expression Vectors. (DOC) [file pone.0021123.s007.doc]

# Table S4

Oligonucleotide Sequences Used for AcGFP- or FLAG-tagged Rab Family Protein Expression Vectors

| **Primer name** | **Sequence (5' to 3')** |
| --- | --- |
| ER1-KZ-AcGFP-For | CCGAATTCGCCACCATGGTGAGCAAGGGCG |
| ER1KzFLG-DDDK9-For ^a^ | CGAATTCGCCACCATGGACTACAAGGATGACGACGACAAG |
| 4KDDD-Nde-Rab11-For ^a^ | GGATGACGACGACAAGCATATGGGCACCCGCGACGACGAGTA |
| hRab11a-Eco1Bam-Rev | CCGGATCCGAATTCTTAGATGTTCTGACAGCACTGCACC |
| BamSV40proNeo-For ^b^ | GAGGATCCAGGCAGGCAGAAGTATGCAAAG |
| BamSV40polyANeo-Rev ^b^ | GAGGATCCAGACATGATAAGATACATTGA |
| Kz-AcGFP-for ^c^ | GCCACCATGGTGAGCAAGGGCGCCGAGCTG |
| AcGFP-XhoER5NheG-rev ^c^ | CGCTAGCGATATCCTCGAGATCTGAGTCCGGACTT |

^a^ These forward primers were used as a mixture.

^b^ These primers were used for amplification of the expression cassette of neomycin resistance gene.

^c^ These primers were 5'-phospholyrated and used for amplification of the AcGFP-MCS fragment.
