# Supplementary material for: Apical Transport of Influenza A Virus Ribonucleoprotein Requires Rab11-positive Recycling Endosome
Source: PLoS One. 2011 Jun 22;6(6):e21123. doi: 10.1371/journal.pone.0021123 (PMC3120830; doi:10.1371/journal.pone.0021123)
Supplement: Table S5 — Oligonucleotide Sequences Used for the Construction of Rab11-FIPs Deletion Mutant Expression Vectors. (DOC) [file pone.0021123.s008.doc]

# Table S5

Oligonucleotide Sequences Used for the Construction of Rab11-FIPs Deletion Mutant Expression Vectors.

| **Primer name** | **Sequence (5' to 3')** |
| --- | --- |
| Xho-KZ-mSB-For | CGACTCGAGCCACCATGGTGAGCAAGGGCG |
| STP-Nhe-mSB-Rev | CTAGCTAGCCTTGTACAGCTCGTCCATGCC |
| Nhe-Nde-FIP1RBD-For | ATCGCTAGCCATATGAGCCCCTCGGACCCTGCATT |
| FIP1RBD-STP-Bam-Rev | ATCGGATCCTTACATCTTTCCTGCTTTTTTGCCA |
| Nhe-Nde-FIP2RBD-For | ATCGCTAGCCATATGGCAGGGTATCGTAGTCTGAC |
| FIP2RBD-STP-Bam-Rev | ATCGGATCCTTAACTGTTAGAGAATTTGCCAGCTT |
| Nhe-Nde-FIP3RBD-For | ATCGCTAGCCATATGAGCATCCAGGGCGCCAAGAG |
| FIP3RBD-STP-Bam-Rev | ATCGGATCCCTACTTGACCTCCAGGATGGACGG |
| Nhe-Nde-FIP4RBD-For | ATCGCTAGCCATATGGGGCAGATTTTGAGCCTCAG |
| FIP4RBD-STP-Bam-Rev | ATCGGATCCTTAGTGTTTGATCTCGAGGATGG |
| Nhe-Nde-FIP5RBD-For | ATCGCTAGCCATATGGCCCCCCAGGCTGGCCAGAT |
| FIP5RBD-STP-Bam-Rev | ATCGGATCCCTATTTGGGGGGGCCCGGGGGGATCT |
| Nhe-FLAG-ERV_Sense ^a^ | CTAGCGACTACAAGGATGACGACGACAAGTAGAT |
| Nhe-FLAG-ERV_Antisence ^a^ | ATCTACTTGTCGTCGTCATCCTTGTAGTCG |
| XhoKz-FIP1B-For | CGACTCGAGCCACCATGTCCCTAATGGTCTCGGCT |
| FIP1woRBDSTP-Nhe-Rev | ATCGCTAGCCTCGCGGACCTGGAACTCCTTCTTGC |
| XhoKz-FIP2-For | CGACTCGAGCCACCATGATGCTGTCCGAGCAAGCC |
| FIP2woRBDSTP-Nhe-Rev | ATCGCTAGCTTCCCGGATGTGGGTGTCTTTCCTCC |
| XhoKz-FIP3-For | CGACTCGAGCCACCATGGCGTCGGCCCCGCCGGCC |
| FIP3woRBDSTP-Nhe-Rev | ATCGCTAGCGCGGAAGTTGATCTCCTCCTGCTTCT |
| XhoKz-FIP4-For | CGACTCGAGCCACCATGGCGGGCGGCGCGGGCTGG |
| FIP4woRBDSTP-Nhe-Rev | ATCGCTAGCCCGGAAGTTGATCTCCTCCTGCTCCT |
| XhoKz-FIP5-For | CGACTCGAGCCACCATGGCCCTGGTGCGGGGCGCG |
| FIP5woRBDSTP-Nhe-Rev | ATCGCTAGCCTCCTGCACATGCTCGTCCCGCTGGC |

^a^ These oligo DNAs were annealed and used as a FLAG cassette.
