# Supplementary material for: Apical Transport of Influenza A Virus Ribonucleoprotein Requires Rab11-positive Recycling Endosome
Source: PLoS One. 2011 Jun 22;6(6):e21123. doi: 10.1371/journal.pone.0021123 (PMC3120830; doi:10.1371/journal.pone.0021123)
Supplement: Table S6 — Oligonucleotide Sequences Used for Polarity-specific Reverse Transcription and Segment-specific Semiquantitative PCR. (DOC) [file pone.0021123.s009.doc]

**Table S6**

Oligonucleotide Sequences Used for Polarity-specific Reverse Transcription and Segment-specific Semiquantitative PCR.

| **Primer name ^a^** | **Sequence (5' to 3') ^b^** |
| --- | --- |
| qRTPR8-PB2F | GAGAGAGAACTGGTCCGCAAAACG |
| qRTPR8-PB2R | TCATCATTCCTCACTTCCCCTCCTG |
| qRTPR8-PB1F | TATTAGGCGTCTCCATCCTGAATCTTG |
| qRTPR8-PB1R | AACCTGTCGACTCCGGCTTGAATC |
| qRTPR8-PAF | GAAATCACAGGAACAATGCGCAAGC |
| qRTPR8-PAR | TGGACATTTGAGACAGCTTGCCCTC |
| qRTPR8-HAF | GGCATCATCACCTCAAACGCATC |
| qRTPR8-HAR | ATCCTCAATTTGGCACTCCTGACG |
| qRTPR8-NPF | TGGCGCCAAGCTAATAATGGTGAC |
| qRTPR8-NPR | ACCTTGCATCAGAGAGCACATCCTG |
| qRTPR8-NAF | ATGTTCCTGTTACCCTGATACCGGC |
| qRTPR8-NAR | TTGTCACCGAAAACCCCACTGC |
| qRTPR8-MF | AGAGGGAGATAACATTCCATGGGGC |
| qRTPR8-MR | TGTTCACAGGTTGCACATACCAGGC |
| qRTPR8-NSF | GAAAGTGGCAGGCCCTCTTTGTATC |
| qRTPR8-NSR | TCGCCAACAATTGCTCCCTCTTC |

^a^ Eight forward primers (-F) and eight reverse primers (-R) were mixed and used for a reverse transcription of negative- and positive-sense viral RNAs, respectively.

^b^ Sequences were designed based on an influenza virus A/Puerto Rico/8/34 (PR8) strain which has been maintaining in our laboratory and, hence, we are not able to guarantee the compatibility of these primers to use for another lot or strain of influenza virus without any modification.
