# Supplementary material for: Apical Transport of Influenza A Virus Ribonucleoprotein Requires Rab11-positive Recycling Endosome
Source: PLoS One. 2011 Jun 22;6(6):e21123. doi: 10.1371/journal.pone.0021123 (PMC3120830; doi:10.1371/journal.pone.0021123)
Supplement: Table S7 — Mean and Maximum Velocities and Migration Lengths of Individual Motile Events. (DOC) [file pone.0021123.s010.doc]

**Table S7**

Mean and Maximum Velocities and Migration Lengths of Individual Motile Events.

| **Event No.** | **Track No.^a^** | **Start Time ^b^ [s]** | **End Time ^b^ [s]** | **Length [μm]** | **Vmean [μm/s]** | **Vmax [μm/s]** |
| --- | --- | --- | --- | --- | --- | --- |
| 1 | 1 | 7.50 | 10.50 | 5.69 | 1.90 | 3.64 |
| 2 | 2 | 0.25 | 1.50 | 1.73 | 1.38 | 2.51 |
| 3 | 2 | 1.50 | 2.50 | 1.52 | 1.52 | 2.36 |
| 4 | 4 | 5.75 | 7.00 | 2.68 | 2.14 | 4.32 |
| 5 | 4 | 7.25 | 9.50 | 4.86 | 2.16 | 3.91 |
| 6 | 5 | 11.50 | 13.75 | 2.19 | 0.97 | 1.95 |
| 7 | 5 | 14.00 | 18.25 | 7.48 | 1.76 | 2.47 |
| 8 | 5 | 18.25 | 22.25 | 5.30 | 1.33 | 2.36 |
| 9 | 6 | 0.25 | 2.25 | 4.23 | 2.12 | 3.70 |
| 10 | 7 | 13.75 | 15.75 | 3.31 | 1.65 | 3.09 |
| 11 | 7 | 18.50 | 19.50 | 0.65 | 0.65 | 1.06 |
| 12 | 8 | 15.00 | 16.25 | 2.96 | 2.37 | 4.05 |
| 13 | 9 | 0.25 | 1.75 | 2.02 | 1.35 | 1.88 |
| 14 | 11 | 3.00 | 4.00 | 1.93 | 1.93 | 2.78 |
| 15 | 11 | 4.25 | 5.75 | 3.43 | 2.29 | 5.07 |
| 16 | 11 | 6.00 | 7.25 | 2.62 | 2.09 | 4.53 |
| 17 | 12 | 7.50 | 9.00 | 3.59 | 2.40 | 3.21 |
| 18 | 12 | 9.25 | 10.25 | 2.65 | 2.65 | 4.75 |
| 19 | 13 | 5.75 | 7.50 | 2.09 | 1.20 | 2.11 |
| 20 | 13 | 9.75 | 11.75 | 2.74 | 1.37 | 2.97 |
| 21 | 13 | 13.00 | 15.25 | 2.69 | 1.20 | 2.86 |
| 22 | 14 | 17.25 | 20.00 | 3.62 | 1.31 | 3.25 |
| 23 | 14 | 20.25 | 21.25 | 1.57 | 1.57 | 2.38 |
| 24 | 15 | 22.50 | 24.50 | 3.41 | 1.70 | 2.56 |
| 25 | 16 | 13.25 | 15.00 | 2.88 | 1.64 | 2.51 |
| 26 | 16 | 16.00 | 18.75 | 3.96 | 1.44 | 3.92 |
| 27 | 16 | 19.00 | 21.50 | 3.11 | 1.24 | 2.54 |
| 28 | 17 | 13.25 | 15.25 | 5.59 | 2.80 | 6.11 |
| 29 | 18 | 17.50 | 19.25 | 3.27 | 1.87 | 3.27 |
| 30 | 18 | 19.50 | 22.25 | 6.45 | 2.35 | 2.99 |
| 31 | 19 | 0.25 | 1.50 | 1.10 | 0.88 | 1.89 |
| 32 | 19 | 1.75 | 5.25 | 3.93 | 1.12 | 3.02 |
| 33 | 20 | 2.50 | 4.25 | 1.33 | 0.76 | 1.46 |
| 34 | 20 | 7.50 | 8.50 | 0.46 | 0.46 | 1.07 |
| 35 | 20 | 8.50 | 12.75 | 5.09 | 1.20 | 3.22 |
| 36 | 21 | 8.75 | 11.25 | 3.17 | 1.27 | 2.46 |
| 37 | 21 | 12.50 | 14.00 | 2.69 | 1.80 | 2.96 |
| 38 | 22 | 7.75 | 11.00 | 3.24 | 1.00 | 1.53 |
| 39 | 23 | 10.25 | 13.50 | 3.93 | 1.21 | 2.90 |
| 40 | 24 | 14.00 | 15.25 | 2.13 | 1.71 | 2.27 |
| 41 | 25 | 3.50 | 6.00 | 4.26 | 1.70 | 3.10 |
| 42 | 26 | 9.50 | 11.50 | 2.25 | 1.13 | 2.17 |
| 43 | 26 | 11.50 | 12.75 | 1.67 | 1.34 | 3.04 |
| 44 | 27 | 22.50 | 24.75 | 2.61 | 1.16 | 2.57 |
| 45 | 28 | 3.00 | 5.50 | 6.32 | 2.53 | 4.81 |
| 46 | 29 | 0.25 | 1.25 | 0.61 | 0.61 | 0.87 |
| 47 | 29 | 1.50 | 3.50 | 2.10 | 1.05 | 2.11 |
| 48 | 30 | 3.00 | 4.50 | 3.00 | 2.00 | 3.65 |
| 49 | 31 | 7.25 | 8.25 | 2.53 | 2.53 | 4.07 |
| 50 | 32 | 10.00 | 12.00 | 3.05 | 1.52 | 3.48 |
| 51 | 33 | 10.50 | 12.50 | 2.63 | 1.32 | 3.52 |
| 52 | 35 | 8.75 | 11.00 | 3.62 | 1.61 | 3.97 |
| 53 | 36 | 18.00 | 20.25 | 3.95 | 1.76 | 2.99 |
| 54 | 38 | 15.00 | 18.50 | 5.34 | 1.53 | 3.13 |
| 55 | 39 | 16.00 | 17.25 | 2.13 | 1.70 | 3.02 |
| 56 | 40 | 0.25 | 1.25 | 1.99 | 1.99 | 2.70 |
| 57 | 40 | 1.50 | 3.00 | 1.93 | 1.29 | 2.93 |
| 58 | 41 | 4.50 | 6.00 | 1.94 | 1.29 | 3.08 |
| 59 | 41 | 6.25 | 7.75 | 1.74 | 1.16 | 2.71 |
| 60 | 41 | 8.25 | 11.25 | 6.72 | 2.24 | 3.47 |
| 61 | 41 | 11.25 | 12.25 | 1.94 | 1.94 | 3.26 |
| 62 | 41 | 13.25 | 14.25 | 1.36 | 1.36 | 3.45 |
| 63 | 42 | 14.75 | 16.50 | 2.74 | 1.56 | 3.04 |
| 64 | 43 | 6.50 | 7.75 | 0.67 | 0.53 | 0.80 |
| 65 | 43 | 11.25 | 13.50 | 2.63 | 1.17 | 1.95 |
| 66 | 43 | 14.25 | 15.50 | 1.87 | 1.50 | 2.04 |
| 67 | 44 | 19.50 | 20.50 | 2.32 | 2.32 | 4.03 |
| 68 | 44 | 21.25 | 22.50 | 1.67 | 1.33 | 2.32 |
| 69 | 45 | 13.25 | 14.25 | 0.42 | 0.42 | 0.60 |
| 70 | 45 | 17.00 | 18.50 | 1.73 | 1.15 | 1.53 |
| 71 | 45 | 20.25 | 22.75 | 3.26 | 1.31 | 2.44 |
| 72 | 45 | 22.75 | 24.25 | 1.50 | 1.00 | 1.71 |
| 73 | 47 | 9.75 | 10.75 | 2.14 | 2.14 | 3.05 |
| 74 | 47 | 12.25 | 13.50 | 1.05 | 0.84 | 0.99 |
| 75 | 48 | 5.75 | 7.25 | 1.46 | 0.97 | 1.96 |
| 76 | 50 | 3.50 | 5.75 | 2.52 | 1.12 | 2.90 |
| 77 | 52 | 9.00 | 10.75 | 2.65 | 1.52 | 3.59 |
| 78 | 54 | 11.00 | 12.00 | 1.63 | 1.63 | 2.43 |
| 79 | 55 | 4.00 | 5.25 | 1.38 | 1.10 | 2.28 |
| 80 | 56 | 9.00 | 10.00 | 0.94 | 0.94 | 1.65 |
| 81 | 56 | 10.25 | 12.75 | 3.78 | 1.51 | 2.33 |
| 82 | 57 | 0.25 | 1.50 | 1.03 | 0.83 | 1.48 |
| 83 | 57 | 2.50 | 4.25 | 1.33 | 0.76 | 1.14 |
| 84 | 59 | 4.50 | 7.75 | 4.82 | 1.48 | 2.98 |
| 85 | 60 | 6.50 | 9.25 | 6.67 | 2.43 | 5.23 |
| 86 | 61 | 21.25 | 22.75 | 2.75 | 1.83 | 3.34 |
| 87 | 61 | 23.50 | 24.75 | 1.70 | 1.36 | 2.57 |
| 88 | 62 | 4.75 | 6.50 | 1.97 | 1.13 | 1.73 |
| 89 | 63 | 6.25 | 7.75 | 3.00 | 2.00 | 3.17 |
| 90 | 65 | 4.50 | 7.00 | 5.92 | 2.37 | 3.51 |
| 91 | 66 | 14.50 | 16.50 | 1.89 | 0.95 | 2.40 |
| 92 | 67 | 12.25 | 14.25 | 1.96 | 0.98 | 1.68 |
| 93 | 68 | 15.00 | 16.00 | 0.82 | 0.82 | 1.49 |
| 94 | 69 | 0.25 | 2.50 | 2.69 | 1.20 | 2.81 |
| 95 | 70 | 1.25 | 2.25 | 1.46 | 1.46 | 3.48 |
| 96 | 71 | 2.75 | 5.75 | 1.78 | 0.59 | 0.99 |
| 97 | 71 | 7.25 | 8.25 | 0.50 | 0.50 | 0.77 |
| 98 | 71 | 13.50 | 14.50 | 0.35 | 0.35 | 0.56 |
| 99 | 71 | 15.00 | 16.00 | 0.36 | 0.36 | 0.57 |
| 100 | 71 | 17.00 | 18.25 | 1.17 | 0.94 | 1.15 |
| 101 | 71 | 23.75 | 24.75 | 1.03 | 1.03 | 2.00 |
| 102 | 72 | 17.00 | 19.25 | 4.21 | 1.87 | 3.77 |
| 103 | 73 | 1.00 | 2.00 | 0.81 | 0.81 | 1.11 |
| 104 | 73 | 2.00 | 3.00 | 2.30 | 2.30 | 2.98 |
| 105 | 74 | 2.75 | 4.50 | 3.66 | 2.09 | 2.84 |
| 106 | 74 | 4.75 | 6.00 | 2.57 | 2.06 | 4.13 |
| 107 | 74 | 9.00 | 11.75 | 6.66 | 2.42 | 5.25 |
| 108 | 75 | 20.25 | 22.75 | 3.05 | 1.22 | 3.00 |
| 109 | 75 | 22.75 | 24.00 | 2.33 | 1.86 | 4.01 |
| 110 | 76 | 8.50 | 9.75 | 2.71 | 2.17 | 2.69 |
| 111 | 77 | 17.25 | 18.75 | 3.67 | 2.45 | 4.42 |
| 112 | 78 | 0.25 | 6.75 | 5.79 | 0.89 | 2.81 |
| 113 | 79 | 9.25 | 10.50 | 1.95 | 1.56 | 3.01 |
| 114 | 80 | 12.50 | 13.50 | 0.64 | 0.64 | 0.81 |
| 115 | 80 | 13.50 | 15.75 | 1.97 | 0.87 | 1.96 |
| 116 | 80 | 16.00 | 18.75 | 1.66 | 0.60 | 1.32 |
| 117 | 81 | 12.75 | 14.25 | 2.31 | 1.54 | 2.37 |
| 118 | 84 | 8.50 | 9.50 | 1.25 | 1.25 | 2.01 |
| 119 | 85 | 1.50 | 4.25 | 4.55 | 1.65 | 4.89 |
| 120 | 87 | 2.00 | 4.50 | 2.51 | 1.01 | 2.35 |
| 121 | 87 | 4.75 | 6.25 | 1.50 | 1.00 | 1.54 |
| 122 | 87 | 6.25 | 8.25 | 2.03 | 1.01 | 1.84 |
| 123 | 90 | 11.75 | 12.75 | 1.32 | 1.32 | 1.61 |

^a^ Track numbers were indicated on Video S1 and Figure 1A (most right image).

^b^ Elapsed time indicated on each image of Video S1.
