# Supplementary material for: Apical Transport of Influenza A Virus Ribonucleoprotein Requires Rab11-positive Recycling Endosome
Source: PLoS One. 2011 Jun 22;6(6):e21123. doi: 10.1371/journal.pone.0021123 (PMC3120830; doi:10.1371/journal.pone.0021123)
Supplement: Materials and Methods S1 — Details of the antibodies utilized in this study and methods for DNA construction, establishment of cell lines, and immunofluorescent microscopy were described. (DOC) [file pone.0021123.s014.doc]

# SUPPORTING MATERIALS AND METHODS

# Antibodies

Immunological properties of anti-NP monoclonal antibody (mAb61A5) have been described elsewhere [1]. The mAb61A5 and anti-HA mAb were purified from mouse ascites by ammonium sulfate precipitation. Mouse anti-FLAG M2 mAb (Cat. No. F3165) and rabbit anti-FLAG polyclonal antibody (Cat. No. F7425) were purchased from Sigma-Aldrich, USA. Rabbit anti-Rab11 polyclonal antibody (Cat. No. 71-5300) and non-specific mouse IgG (a component of Zenon Mouse IgG Labeling Kit) were purchased from Life Technologies, USA. Anti-PB2, PB1, PA, and NP antisera were prepared from rabbits individually immunized with recombinant viral proteins. Rabbit anti-NP polyclonal antibody was further purified from the antiserum by affinity chromatography. Rabbit anti-M1 and mouse anti-HA antisera were the kind gifts of Drs. K. Watanabe (Nagasaki University, Japan) and T. Nagai (Kitasato University, Japan), respectively. Peroxidase- and fluorochrome-conjugated antibodies were prepared by using peroxidase labeling kit-NH_2_ (Dojindo Molecular Technologies, Japan) and Alexa Fluor 488/568 (AF488/568) monoclonal antibody labeling kits (Life Technologies) according to the manufacturer's instructions, respectively.

# Construction of plasmid DNA

Complementary DNAs (cDNAs) of human Rab family proteins (Table S1) were generated from total RNAs of human 293T or HeLa cells using specific primers (Table S2). The coding sequence of human α-tubulin in pAcGFP1-Tubulin vector (Clontech Laboratories, USA) was replaced with the human Rab8A, Rab11A, and Rab11B cDNA fragments in-frame at the *Xho* I and *Bam*H I sites (pAcGFP-hRab8A/11A/11B). Dominant negative and constitutively active mutants of human Rab11A (pAcGFP-hRab11A-S25N and -Q70L, respectively) were constructed by inverse PCR-based site-directed mutagenesis using relevant primer set (Table S3). The cDNA of human Rab11A and its mutants, which tagged with the amino-terminal AcGFP or FLAG epitope, were amplified from pAcGFP-hRab11A, -S25N, and -Q70L by PCR using specific primers (Table S4). To construct a protein expression vector pCANeoHA (Figure S1A), the sequence between two *Bam*H I sites of pCHA [2], a derivative of pCAGGS [3], was replaced by the expression cassette of neomycin resistance gene amplified from pcDNA3 (Life Technologies) using specific primers (Table S4). The AcGFP/FLAG-tagged Rab11A cDNAs were cut with *Eco*R I and subcloned between two *Eco*R I sites of pCANeoHA. Other Rab family cDNAs were cloned into pCANeoAcGFP-MCS (Figure S1A), which was constructed by inserting the AcGFP-MCS fragment amplified from pAcGFP1-Tubulin using specific primers (Table S4) between blunt-ended two *Eco*R I sites of pCANeoHA.

The cDNAs of human Rab11 family interacting proteins (Rab11-FIPs) with deletions were similarly amplified using specific primer sets (Table S5). The cDNA encoding monomeric red fluorescent protein (mStrawberry, Clontech Lab.) was subcloned between the *Xho* I and *Nhe* I sites of the pCANeoHA (pCANeoSB). Rab binding domain sequences (FIPnRBD, n=1 to 5) were cloned in-frame between the *Nhe* I and *Eco*R V sites of pCANeoSB. RBD-deleted mutants (FIPnΔRBD, n=1 to 5) and a FLAG sequence cassette were together cloned in-frame between the *Xho* I and *Eco*R V sites of pCANeoHA (Table S5).

# Construction of a standard DNA plasmid for quantitative PCR

Construction scheme was shown in Figure S1B. Target DNA sequences for quantitative PCR (qPCR) were individually amplified from eight distinct segments of influenza virus A/Puerto Rico/8/34 (PR8) cDNA using 5'-phosphorylated (white circles) or non-phosphorylated segment-specific qPCR primer sets (Table S6). Two neighboring DNA fragments (approximately 150 bp), e.g., downstream-phosphorylated fragment 1 and upstream-phosphorylated fragment 2, were ligated and amplified by second PCR using non-phosphorylated primer sets. For concatenation of these DNA fragments, the fragments (approximately 300 bp) were mixed and third PCR was carried out using 5'-phosphorylated qPCR primers for segment 1 upstream (qRTPR8-PB2F) and segment 8 downstream (qRTPR8-NSR). The concatenated target sequence (approximately 1,200 bp) was finally subcloned into the *Eco*R V site of the pBluescript SK(+) plasmid (Agilent Technologies, USA) and the DNA sequence of resultant plasmid (pBSPR8qPCRSTD) was confirmed by DNA sequencing.

# Establishment of stable expression cell lines

Expression vectors of wild type (WT), dominant negative (DN), and constitutively active (CA) mutants of FLAG-tagged Rab11A (pCANeoF-Rab11A, -Rab11A-DN, and -Rab11A-CA, respectively) were transfected to MDCK cells. At 24 h post-transfection, culture medium was replaced by selection medium containing 500 μg/ml of G418. After 1 week, G418-resistant cells were subjected to cell cloning in a 96-well plate. Expression of FLAG-tagged proteins in isolated cells (MDCK-F11A-WT, -DN, and -CA) were confirmed by Western blotting and immunofluorescent analysis using anti-FLAG M2 mAb. Control cell line (MDCK-Neo) and AcGFP-α-Tubulin expressing cell line (MDCK-Tub) were also established with pCANeoHA and pAcGFP1-Tubulin, respectively.

# Immunofluorescence microscopy

MDCK cells were seeded onto a cover glass in a 12-well culture plate (2×10^5^ cells/well) with DMEM (Sigma-Aldrich, Cat. No. D5796) containing 10% fetal bovine serum and antibiotics at 12 h before transfection/infection. DNA transfection was carried out using Lipofectamine 2000 or Lipofectamine LTX with Plus Reagent (Life Technologies) according to the manufacturer’s instructions. At 12 h post-transfection, cells were infected with influenza A virus at multiplicity of infection (moi) of 1 to 3 for 1 h and followed by exchange of the culture medium. Cells were fixed with 4% paraformaldehyde in PBS for 10 minutes, permeabilized with 0.5% Triton X-100 in PBS for 10 minutes, and immersed in a blocking reagent (Blocking One, Nacalai Tesque, Japan). Cells were incubated with primary antibodies and subsequently secondary fluorescent antibodies. Nuclei were counterstained with TO-PRO-3 or DAPI (Life Technologies). Observation was carried out using a confocal laser-scanning system (TCS-NT or TCS-SP5II AOBS, Leica Microsystems, Germany). For polarized MDCK cells, 1.5×10^6^ cells were seeded in a well and the cover glass was mounted by using a 0.1-mm spacer seal.

# REFERENCES

1. Momose F, Kikuchi Y, Komase K, Morikawa Y (2007) Visualization of microtubule-mediated transport of influenza viral progeny ribonucleoprotein. Microbes Infect 9: 1422-1433.

2. Nagata K, Saito S, Okuwaki M, Kawase H, Furuya A, et al. (1998) Cellular localization and expression of template-activating factor I in different cell types. Exp Cell Res 240: 274-281.

3. Niwa H, Yamamura K, Miyazaki J (1991) Efficient selection for high-expression transfectants with a novel eukaryotic vector. Gene 108: 193-199.
